# Comparative metabolomic and transcriptomic analysis reveals a coexpression network of the carotenoid metabolism pathway in the panicle of *Setaria italica*

**DOI:** 10.1186/s12870-022-03467-2

**Published:** 2022-03-08

**Authors:** Hui Li, Shangling Han, Yiqiong Huo, Guifang Ma, Zhaoxia Sun, Hongying Li, Siyu Hou, Yuanhuai Han

**Affiliations:** 1 College of Agriculture, Institute of Agricultural Bioengineering, Shanxi Agricultural University, Taigu, 030801 Shanxi China; 2 Shanxi Key Laboratory of Germplasm Innovation and Molecular Breeding of Minor Crop, Taigu, 030801 Shanxi China

**Keywords:** Foxtail millet, Carotenoid, Transcriptomics, Metabolomics, Coexpression network

## Abstract

**Background:**

The grains of foxtail millet are enriched in carotenoids, which endow this plant with a yellow color and extremely high nutritional value. However, the underlying molecular regulation mechanism and gene coexpression network remain unclear.

**Methods:**

The carotenoid species and content were detected by HPLC for two foxtail millet varieties at three panicle development stages. Based on a homologous sequence BLAST analysis, these genes related to carotenoid metabolism were identified from the foxtail millet genome database. The conserved protein domains, chromosome locations, gene structures and phylogenetic trees were analyzed using bioinformatics tools. RNA-seq was performed for these samples to identify differentially expressed genes (DEGs). A Pearson correlation analysis was performed between the expression of genes related to carotenoid metabolism and the content of carotenoid metabolites. Furthermore, the expression levels of the key DEGs were verified by qRT-PCR. The gene coexpression network was constructed by a weighted gene coexpression network analysis (WGCNA).

**Result:**

The major carotenoid metabolites in the panicles of DHD and JG21 were lutein and β-carotene. These carotenoid metabolite contents sharply decreased during the panicle development stage. The lutein and β-carotene contents were highest at the S1 stage of DHD, with values of 11.474 μg /100 mg and 12.524 μg /100 mg, respectively. Fifty-four genes related to carotenoid metabolism were identified in the foxtail millet genome. Cis-acting element analysis showed that these gene promoters mainly contain ‘plant hormone’, ‘drought stress resistance’, ‘MYB binding site’, ‘endosperm specific’ and ‘seed specific’ cis-acting elements and especially the ‘light-responsive’ and ‘ABA-responsive’ elements. In the carotenoid metabolic pathways, *SiHDS*, *SiHMGS3*, *SiPDS* and *SiNCED1* were more highly expressed in the panicle of foxtail millet. The expression of *SiCMT*, *SiAACT3*, *SiPSY1*, *SiZEP1/2*, and *SiCCD8c/8d* was significantly correlated with the lutein content. The expression of *SiCMT*, *SiHDR*, *SiIDI2*, *SiAACT3*, *SiPSY1*, and *SiZEP1/2* was significantly correlated with the content of β-carotene. WGCNA showed that the coral module was highly correlated with lutein and β-carotene, and 13 structural genes from the carotenoid biosynthetic pathway were identified. Network visualization revealed 25 intramodular hub genes that putatively control carotenoid metabolism.

**Conclusion:**

Based on the integrative analysis of the transcriptomics and carotenoid metabonomics, we found that DEGs related to carotenoid metabolism had a stronger correlation with the key carotenoid metabolite content. The correlation analysis and WGCNA identified and predicted the gene regulation network related to carotenoid metabolism. These results lay the foundation for exploring the key target genes regulating carotenoid metabolism flux in the panicle of foxtail millet. We hope that these target genes could be used to genetically modify millet to enhance the carotenoid content in the future.

**Supplementary Information:**

The online version contains supplementary material available at 10.1186/s12870-022-03467-2.

## Background

Foxtail millet [*Setaria italica* (L.) Beauv.] belongs to the *Setaria* genus of Poaceae grass family, and it is widely planted in Eurasian arid and semiarid areas as a kind of C4 crop that endures drought stress and infertile soils [1]. The ancestor of cultivated foxtail millet is green foxtail grass, which was domesticated early in China at approximately 16,000 years ago according to archaeological evidence [2]. At present, four genome sequences of foxtail millet have been published: ‘Yugu1’, ‘Zhanggu’, ‘*Xiaomi*’ and ‘huagu11’ [3–6]. The construction of a genome database and efficient genetic transformation system lay the foundation for investigating the functional genes, genetic evolution, plant architecture and physiology of foxtail millet, especially as a kind of C4 model and bioenergy grass system [1, 7, 8]. Foxtail millet is an important food crop in China and other Asian countries, and has small grains and higher nutritional value and contains edible fiber, protein, starch, vitamins and mineral elements [9]. Previous studies have shown that dehulled grains of foxtail millet enriched in carotenoids presents a yellow color [10, 11]. Carotenoids, as the main source of vitamin A in the human body, present various functions, such as eyesight protection, antioxidation, and anticancer effects, and they also have preventive functions in a variety of cardiovascular diseases [12]. Biofortified carotenoid food could be essential for meeting the health requirements and reduce “hidden hunger” in developing areas. According to previous reports, the carotenoid content in grains of foxtail millet ranged from 189.1 μg /100 mg to 201.3 μg /100 mg, which are 7.2-, 201.3- and 1.7- times of wheat, brown rice and sorghum [13–15]. Hence, the grains of foxtail millet could be an excellent carotenoid food resource.

The carotenoid metabolism pathway in plants is well known. Carotenoids and their derivatives are composed of isopentenyl pyrophosphate (IPP) and its double bond isomer and dimethylallyl diphosphate (DMAPP). In plant cells, these IPP precursors are mainly synthesized by the mevalonate (MVA) and 2-C-methyl-D-erythritol 4-phosphate (MEP) pathways and the precursors of carotenoid synthesis mainly come from the MEP metabolic pathway [16, 17]. Finally, carotenoid synthesis begins with phytoene synthase (PSY) condensation of two geranylgeranyl pyrophosphate (GGPP) molecules to form a 15 cis isomer. In the next step, all trans-lycopene is produced by phytene saturase (PDS), zeta-e carotene desaturase (ZDS), carotenoid isomerase (Crtiso), and zeta-carotene isomerase (Z-ISO). β-carotene and α-carotene are synthesized by LCYB or LCYE catalysis, and then lutein and zeaxanthin are produced by hydroxylation of nonheme carotene hydroxylase (BCH1 and BCH2) and heme hydroxylase (cytochrome P450-type monooxygenase, CYP97A3 and CYP97C1). Under the catalysis of zeaxanthin cyclooxygenase (ZEP) and pansy xanthine decyclooxygenase (VDE), luteins are produced, including anther xanthine, viola xanthine and neoxanthine [18].

Transcription factors play an important regulatory role in the carotenoid metabolism pathway. Phytochrome-interacting factor 1 (PIF1) regulated for carotenoid biosynthesis by inhibiting the expression of *PSY* in dark environment and also participates in the formation of chloroplasts [19]. An bZIP transcription factor named long hypocotyl 5 (HY5), also take part in carotenoid biosynthesis as a negative regulator of PIF1 during photomorphogenesis [20]. Recently, a sub-clade MADS gene of FRUITFULL transcription factor named CsMADS5 has reported in tomato that it can positively regulate the carotenoid content by up-regulating the expression of *PSY/PDS/LCYB* [21]. So far, some regulators have reported that revolved in carotenoid biosynthesis pathway in the transcript level in plants. However, our understanding of the transcriptional regulation of carotenoid biosynthesis still requires further investigation.

Many previous studies on genes involved in carotenoid biosynthesis and regulated networks in plants have been reported. Through introducing *PSY* and *CRTL* genes in rice, a new edible rice variety named “golden rice” was successfully developed [22]. In maize, they found that the genotype with favorable alleles of *crtRB1* and *lcyE* had a significant effect on the β-carotene content (7.9-fold and 2.1-fold higher) compared to.

the unfavorable genotype [23]. In tomato, two PSY isoforms are responsible for divergent functions of fruit-specific carotenoid accumulation [24]. For foxtail millet, although previous reports on carotenoid components and some key gene expression analyses have been reported, the global analysis of gene expression patterns involved in carotenoid metabolism during the panicle developmental stage has not yet been reported. Therefore, in this study, we proposed to explore the dynamic pattern of carotenoid accumulation and the relationship between carotenoid content and gene expression level during panicle development periods to deeply understand the underlying molecular mechanism of carotenoid metabolism and identify target genes for genetic modification in foxtail millet.

## Methods

### Plant materials

Foxtail millet varieties JG21 and DHD were planted in the experimental field of Shanxi Agricultural University in April 2019 (N: 37°12′, E: 112°28′). The experiments were done in three biological replicates. Samples were collected from the middle part of the panicle at S1 (Beginning of diaspore colouring, 111 days after imbibition), S2 (Colouring of half of diaspores, 129 days after imbibition) and S3 (Colouring of almost all diaspores, 143 days after imbibition) (Fig. S1) [25]. For each cultivar, the middle of panicles were collected and mixed in one tube from the 10 independent plants. All samples were frozen and stored at - 80 °C for following analysis. (Study complied with local and national regulations for using plants.)

### Extraction and determination of carotenoids

The extraction of carotenoids followed Paul’s method [26]. In brief, a fine powder generated from each sample (approximately 0.5 g) was added to 40 μL 50% KOH solution and 2 mL anhydrous ethanol with 0.1% BHT (butylated hydroxytoluene). After vortex mixing, the samples were bathed in water at 85 °C for 5 min, made up volume with 1 mL cold water. Then, 1 ml n-hexane was added and centrifuged at 5000 g for 5 min after vortexing. The supernatant was removed and transferred to a new centrifuge tube. Then, 1 mL of ultrapure water was added to the final n-hexane phase, which was vortexed and centrifuged, and then the supernatant was dried with nitrogen until it became dry matter. Finally, it was dissolved in 200 μL of acetonitrile: ethanol (1:4) added with 0.1% BHT.

The chromatographic system was a DGLC dual ternary ultrahigh-performance liquid chromatography system (Thermo, USA). A YMC Carotenoid S-3 μm (150*4.6 mm) liquid chromatographic column was used. The injection volume was 2 μl, the column temperature was 40 °C, and the detection wavelength was 450 nm. The solvent systems contained mobile phase A (methanol: water (1:1)) and mobile phase B (acetonitrile (ACN): ethyl acetate (3:1)). All solvents used were HPLC grade and filtered through a 0.2-mm filter prior to use. The gradient was 30% A:70% B for 0.5 min, then increased in intervals to 0.1% A:99.9% B for 5.5 min and then to 30% A:70% B for the last 2 min. UPLC–MS/MS was performed by Sanshu Biotechnology Co., Ltd. (ShangHai, China) (Fig. S2).

### Retrieval gene sequence, collinear analysis and chromosome location

The genes involved in the map00900 and map00906 metabolic pathways were analyzed and mapped to Kyoto Encyclopedia of Genes and Genomes Database (KEGG, https://www.kegg.jp/). The sequences of key genes in the carotenoid metabolism pathway of foxtail millet were obtained from homozygous genes in *Arabidopsis thaliana*. TBtools analysis tools were used for the gene sequence information analysis, collinearity analysis, and chromosome mapping analysis [27]. The online websites were used for protein domain prediction analysis (http://pfam.xfam.org/search/) and subcellular location prediction (https://wolfpsort.hgc.jp/).

### RNA extraction and RNA-seq analysis

The developing panicle of DHD and JG21 were separated at the S1/S2/S3 filling stages, and total RNA was isolated using a Quick RNA Isolation kit (Takara Corporation, Dalian, China) [28]. RNA quality and concentration were assessed by 1% agarose electrophoresis (electrophoresis on a denaturing agarose gel) and a NanoPhotometer® spectrophotometer (IMPLEN, CA, USA). The library construction of qualified RNA samples was carried out with a target insert size of ~ 450 bp, and the quality of the RNA-seq libraries was evaluated by an Agilent Bioanalyzer 2100 system (Agilent Technologies, CA, USA). After the library profile analysis, the RNA-seq libraries were sequenced on an Illumina HiSeq platform following standard methods by Novogene Life Sciences Pvt. Ltd. Beijing, China, with three biological replicates, and 150 bp paired-end reads were generated. After filtering the raw data, clean reads were mapped to the foxtail millet reference genome (https://phytozome.jgi.doe.gov/pz/portal.html#!info?alias=Org_Sitalica) using Hisat2 (http://ccb.jhu.edu/software/hisat2/index.shtml) [29].

For gene expression quantification, HTSeq was used to count the read numbers mapped to each gene, and the FPKM (fragments per kilobase per million fragments) value was used to normalize the expression level of each gene. DEGs between different samples were identified using the R package DESeq with |log2FoldChange| > 1 and *P* value < 0.05 as the thresholds [30]. Volcano and MA plots of DEGs were drawn by the R package ggplot2. The KEGG (Kyoto Encyclopedia of Genes and Genomes, http://www.genome.jp/kegg) and GO (Gene Ontology, http://geneontology.org/) annotations of DEGs were further performed with GOseq and KOBAS software, respectively [31]. The FPKM values of key genes involved in the carotenoid synthesis pathway at different developmental stages of ‘DHD’ and ‘JG21’ spikelets were obtained, and the correlation coefficients between the FPKM values of candidate genes and carotenoid content were calculated using SPSS 19.0 software (SPSS, Inc., Chicago, IL, USA). Heat maps of gene expression and correlation coefficients were drawn by TBtools [27]. A weighted gene coexpression network analysis of all genes was performed using the R package WGCNA. The selection of interacting genes used to construct a coexpression network is based on the following principles: 1. The modules correlated with the main carotenoid components were selected according to the correlation coefficient over 0.9 between module eigengene and specific carotenoid compound. 2. Meantime, in the correlated module, searching the carotenoid metabolism pathway genes, and extracted the top 25 transcription factors with the coexpression weight over 0.25. 3. Hub genes related to carotenoid metabolites and 25 coexpressed transcription factors were further selected to construct a coexpression network. 4.Whether transcription factors can combine the binding sites in the upstream of the promoter of carotenoid metabolism genes were predicted by using the FIMO (Find Individual Motif Occurences) tools (https://meme-suite.org/meme/tools/fimo) according to default parameters.

### qRT-PCR analysis

qRT-PCR was performed to verify the expression patterns revealed by the RNA-seq study. Total RNA samples of three stages of foxtail millet panicles were extracted using TRIzol reagent (Invitrogen, Carlsbad, CA, USA). Purified RNA samples were reverse-transcribed using the PrimeScript RT Reagent Kit with gDNA Eraser (Takara, Dalian, China) according to the manufacturer’s protocol. Four transcripts were selected for the qRT-PCR assay. Gene-specific qRT-PCR primers were designed using Primer 3 software (http://primer3.ut.ee/) (Table S1). qRT-PCR was carried out using a Bio–Rad CFX96 instrument (Bio–Rad Laboratories, USA). Each reaction mix was composed of 10 μl 2 × SYBR Green Master Mix Reagent (Vazyme Biotech, China), 2.0 μl cDNA sample, and 400 nm gene-specific primers in a final volume of 20 μl PCR conditions were as follows: 2 min at 95 °C, followed by 40 cycles of denaturation at 95 °C for 10 s and annealing at 60 °C for 40 s. The relative mRNA level for each gene was calculated using the 2^−∆∆CT^ formula [32].

## Results

### Dynamic changes in carotenoid metabolites during panicle development stages

**Fig. 1:**
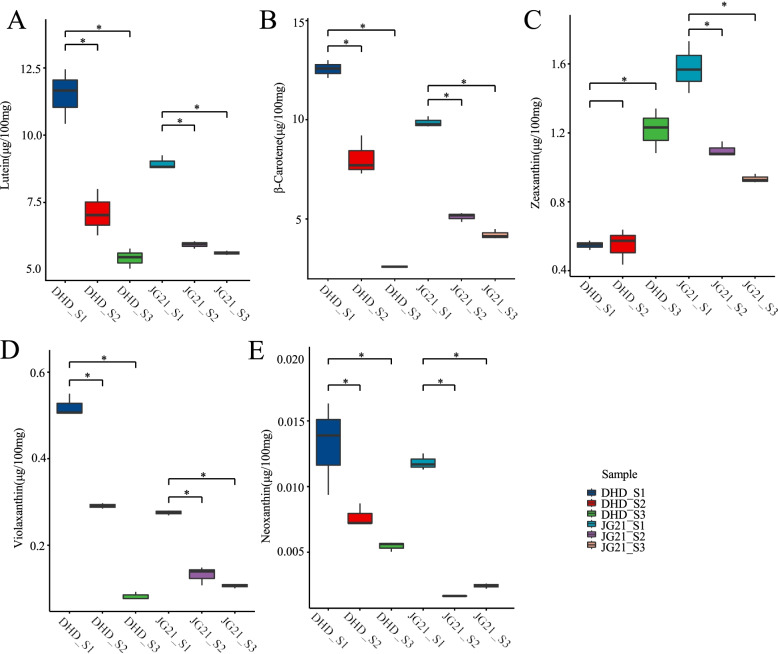
DHD and JG21 carotenoid content at the three developmental stages of panicle. (**A**) Lutein. (**B**) β-carotene. (**C**) Zeaxanthin. (**D**) Violaxanthin. (**E**) Neoxanthin

For two foxtail millet cultivars, five carotenoid metabolites, lutein, β-carotene, zeaxanthin, violaxanthin, and neoxanthin, were detected in the spikes at the three panicle development stages by HPLC. The results showed that most of carotenoid metabolite contents were higher at the S1 stage than at the other stages of the two cultivars except zeaxanthin of DHD (Fig. 1). The first major carotenoid metabolite was lutein, accounting for 43.28 to 58.18% of the total carotenoid content. The second major carotenoid metabolite was β-carotene, accounting for 27.82 to 50.36% of the total carotenoid content (Fig. S3). Moreover, we found that the contents of two major carotenoid (Lutein and β-carotene) metabolites in JG21 were lower than those in DHD at the S1&S2 but became higher at S3. The highest lutein and β-carotene contents of 11.474 μg /100 mg and 12.524 μg /100 mg, respectively, were observed at the S1 stage of DHD.

### Sequence characteristics of genes related to the carotenoid metabolism pathway

**Fig. 2:**
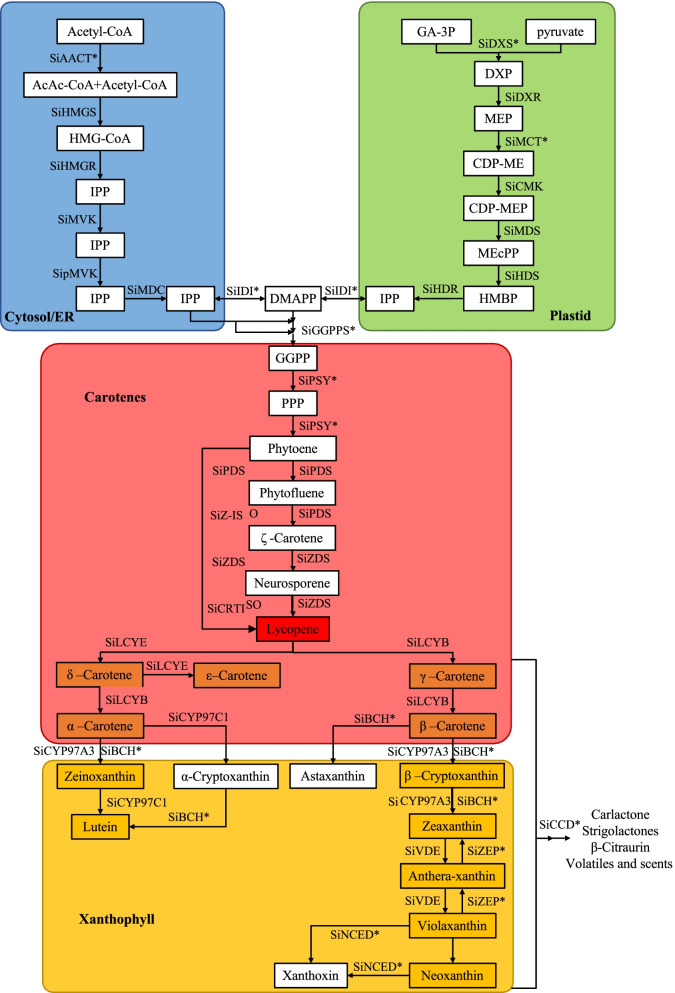
Foxtail millet carotenoid metabolism pathway

**Fig. 3:**
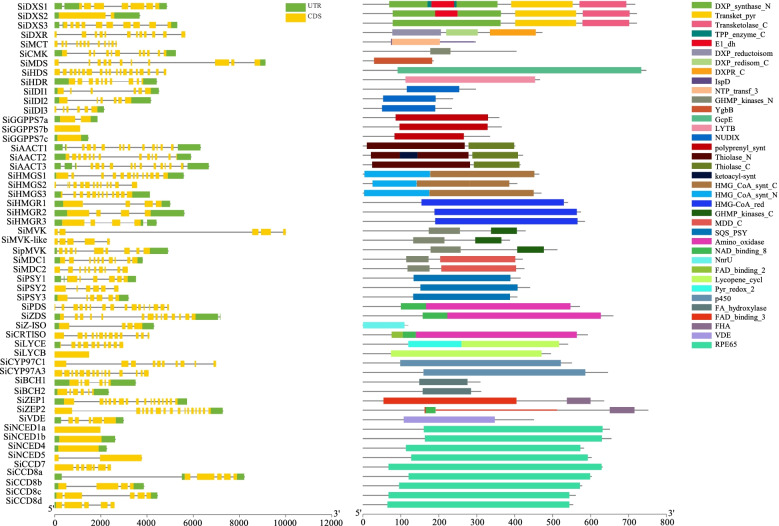
Gene structure and protein structure of the carotenoid metabolism pathway infoxtail millet

Based on the homologous protein blast method, fifty-four genes related to carotenoid metabolism were identified from the genome database (Table 1). Of them, fifteen and fourteen genes encoded six and nine key isozymes involved in the MVA and MEP metabolism pathways (Fig. 2), respectively. The remaining genes encoded fifteen enzymes involved in the carotenoid biosynthesis and degradation pathways. The protein lengths of these genes ranged from 233 ~ 751 amino acid residues. The protein molecular weights of these genes ranged from 26,535 ~ 82,255 Da. Moreover, 34 genes could be targeted to chloroplasts according to the protein prediction of subcellular localization. The remaining 2, 6, 4, 4 and 2 genes could be targeted to the cytoskeleton, endoplasmic reticulum, mitochondrion, nucleus and plasma membrane, respectively. The *SiGGPPS7b, SiLCYB* and *SiNNCED4* gene structures did not have any introns. The shortest gene genomic sequence length was *SiGGPPS7b* at 1098 bp. The longest gene genomic sequence length was *SiCYP97C1* at 18373 bp. These homologous genes for each gene family contained the same specific conserved protein domain (Fig. 3).

**Table 1:** Carotenoid metabolism gene name, ID, and sequence information

Gene	E.C.	NCBI ID	***Xiaomi*** ID	yugu1 ID	isoelectric point	molecule weight	PSORT	protein length
SiDXS1	2.2.1.7	101,752,673	Si3g23880	Seita.3G245500	6.8	77,109.14	cyto	717
SiDXS2	2.2.1.7	101,761,672	Si2g07510	Seita.2G068200	6.85	76,986.96	mito	721
SiDXS3	2.2.1.7	101,755,270	Si4g03680	Seita.4G031100	6.01	78,989.42	chlo	721
SiDXR	1.1.1.267	101,779,357	Si5g07910	Seita.5G071800	6.44	51,245	cysk	472
SiMCT	2.7.7.60	101,776,634	Si5g40650	Seita.5G407200	8.2	32,333.52	chlo	297
SiCMK	2.7.1.148	101,762,054	Si5g35430	Seita.5G351100	6.04	43,879.51	chlo	404
SiMDS	4.6.1.12	101,757,872	Si1g27830	Seita.1G272600	9.52	65,443.67	nucl	605
SiHDS	1.17.7.1	101,757,740	Si1g23200	Seita.1G223900	5.63	82,255.08	cyto	746
SiHDR	1.17.7.4	101,779,322	Si9g10620	Seita.9G105600	5.64	51,719.51	chlo	466
SiIDI1	5.3.3.2	101,776,451	Si3g23600	Seita.3G241900	5.94	32,873.71	chlo	297
SiIDI2	5.3.3.2	101,757,504	Si2g34540	Seita.2G344000	6.28	37,523.76	mito	334
SiIDI3	5.3.3.2	101,757,120	Si2g34530	Seita.2G343900	5.19	26,535.25	cyto	233
SiGGPPS7a	2.5.1.1	101,763,054	Si2g36780	Seita.2G368100	6.18	38,272.95	mito	359
SiGGPPS7b	2.5.1.1	101,781,099	Si5g07040	Seita.5G062900	6.46	37,432.52	chlo	365
SiGGPPS7c	2.5.1.1	101,773,288	Si1g27280	Seita.1G266500	6.15	35,656.98	chlo	340
SiAACT1	2.3.1.9	101,762,107	Si7g28520	Seita.7G280000	6.02	41,059.23	chlo	401
SiAACT2	2.3.1.9	101,752,713	Si5g08110	Seita.5G074000	8.11	43,122.41	chlo	421
SiAACT3	2.3.1.9	101,771,647	Si5g31350	Seita.5G308600	5.83	41,231.32	chlo	401
SiHMGS1	2.3.3.10	101,757,046	Si9g54750	Seita.9G564900	6.1	70,931.78	nucl	649
SiHMGS2	2.3.3.10	101,761,667	Si6g23300	Seita.6G234800	6.11	48,769.71	nucl	436
SiHMGS3	2.3.3.10	101,778,463	Si2g28300	Seita.2G279400	5.82	52,344.33	nucl	470
SiHMGR1	1.1.1.34	101,777,901	Si1g31130	Seita.1G294900	9.02	56,290.87	plas	540
SiHMGR2	1.1.1.34	101,754,923	Si6g20840	Seita.6G208200	7.94	60,576.86	E.R.	574
SiHMGR3	1.1.1.34	101,777,103	Si2g26210	Seita.2G257000	8.35	62,101.51	E.R.	584
SiMVK	2.7.1.36	101,779,972	Si3g26580	Seita.3G273700	5.42	40,498.61	plas	387
SiMVK-like	2.7.1.36	101,761,036	Si2g35580	Seita.2G354500	5.34	40,499.55	cyto	387
SipMVKp	2.7.4.2	101,757,567	Si9g45700	Seita.9G467300	6.19	54,814.23	cyto	512
SiMDC1	4.1.1.33	101,783,413	Si1g35390	Seita.1G351000	5.96	46,029.43	chlo	420
SiMDC2	4.1.1.33	101,785,083	Si3g38700	Seita.3G395300	6.03	46,365.95	cyto	423
SiPSY1	2.5.1.32	101,786,849	Si4g27520	Seita.4G288600	8.97	46,899.94	chlo	415
SiPSY2	2.5.1.32	101,756,152	Si2g30580	Seita.2G303000	9.04	48,655.63	chlo	440
SiPSY3	2.5.1.32	101,759,707	Si3g38930	Seita.3G397800	8.78	45,494.92	chlo	409
SiPDS	1.3.5.5	101,771,481	Si9g50120	Seita.9G515900	8.38	69,490.07	chlo	619
SiZDS	1.3.5.6	101,786,776	Si2g08440	Seita.2G077800	7.98	63,378.75	chlo	575
SiZ-ISO	5.2.1.12	101,783,502	Si3g30280	Seita.3G304800	9.3	40,735.41	chlo	373
SiCRTISO	5.2.1.13	101,781,301	Si8g16170	Seita.8G158400	6.3	64,075.75	chlo	592
SiLYCE	5.5.1.18	101,764,899	Si5g21910	Seita.5G258300	6.24	50,077.69	cyto	442
SiLYCB	5.5.1.19	101,763,950	Si1g06300	Seita.1G055200	7.18	53,665.03	chlo	495
SiCYP97C1	1.14.14.158	101,758,095	Si9g33310	Seita.9G336100	5.92	65,129.85	chlo	583
SiCYP97A3	1.14.-.-	101,780,326	Si1g36810	Seita.1G367300	5.88	70,177.46	chlo	645
SiBCH1	1.14.15.24	101,770,294	Si9g54300	Seita.9G559200	9.12	33,335.42	chlo	309
SiBCH2	1.14.15.24	101,757,118	Si7g21990	Seita.7G209000	9.83	33,887.3	chlo	311
SiZEP1	1.14.15.21	101,781,949	Si7g13140	Seita.7G116800	7.16	69,731.49	mito	635
SiZEP2	1.14.15.21	101,780,465	Si7g13100	Seita.7G116400	8.82	82,101.33	chlo	751
SiVDE	1.23.5.1	101,754,003	Si7g08440	Seita.7G067200	5.32	51,047.11	chlo	450
SiNCED1a	1.13.11.51	101,783,411	Si1g31780	Seita.1G288400	6.33	70,862.24	chlo	659
SiNCED1b	1.13.11.51	101,778,945	Si9g15380	Seita.9G156500	6.08	65,918.88	chlo	607
SiNCED4	1.13.11.51	101,766,978	Si2g04470	Seita.2G035400	6.3	62,697.22	chlo	582
SiNCED5	1.13.11.51	101,770,668	Si3g38270	Seita.3G391000	6.56	70,850.96	chlo	659
SiCCD7	1.13.11.68	101,764,126	Si7g20330	Seita.7G189300	8.9	67,763.96	chlo	619
SiCCD8a	1.13.11.69	101,774,274	Si8g10360	Seita.8G101900	7.91	59,808.24	chlo	542
SiCCD8b	1.13.11.69	101,767,759	Si5g32040	Seita.5G315800	6.48	62,634.96	chlo	577
SiCCD8c	1.13.11.69	101,763,948	Si5g32020	Seita.5G315600	6.04	61,291.42	chlo	560
SiCCD8d	1.13.11.69	101,767,361	Si5g32030	Seita.5G315700	7.66	60,585.91	chlo	553

**Fig. 4:**
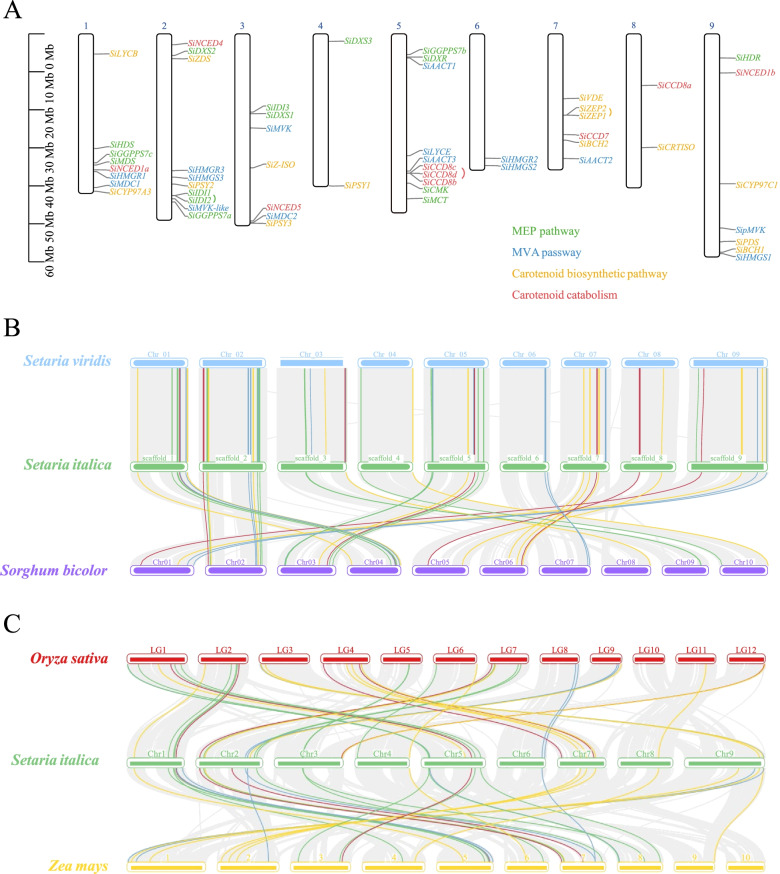
(A) Subcellular location of carotenoid metabolism genes. (**B**) Collinearity analysis of millet carotenoid metabolism genes in close-source species. (**C**) Collinearity analysis of millet carotenoid metabolism genes inthemain monocotcrops

Fifty-four genes related to the carotenoid metabolism pathway were distributed on nine chromosomes of foxtail millet. Eight genes were mapped on chromosome 1, which were *SiLYCB/SiHDS/SiGGPPS7c/SiMDS/SiNCED1a/SiHMGR1/SiMDC1/SiCYP97A3.* Ten genes were mapped on chromosome 2, which were *SiNCED4/SiDXS2/SiZDS/SiHMGR3/SiHMGS3/SiPSY2/SiIDI1/SiIDI2/SiMVK-like/SiGGPPS7a*. Seven genes were mapped on chromosome 3, which were *SilDI3/SiDXS1/SiMVK/SiZ-ISO/SiNCED5/SiMDC2/SiPSY3*. Two genes were mapped on chromosome 4, which were *SiDXS3* and *SiPSY1*. Ten genes were mapped on chromosome 5, which were *SiGGPPS7b/SiDXR/SiAACT1/SiLYCE/SiAACT3/SiCCD8c/SiCCD8b/SiCMK/SiMCT*. Two genes were mapped on chromosome 6, which were *SiHMGR2* and *SiHMGS2*. Six genes were mapped on chromosome 7, which were *SiVDE/SiZEP2/SiZEP1/SiCCD7/SiBCH2/SiAACT2*. Two genes were mapped on chromosome 8, which were *SiCCD8a* and *SiCRTISO*. Finally, seven genes were mapped on chromosome 9, which were *SiHDR/SiNCED1b/SiCYP97C1/SipMVK/SiPDS/SiBCH1/SiHMGS1*. A genomic collinearity analysis showed 53, 46, 33, and 37 homologous genes in the *S. viridis*, *S. bicolor*, *Z. mays* and *O. sativa* genomes, respectively, compared with the *S. italica* genome (Fig. 4).

Phylogenetic trees of homologous proteins related to carotenoid metabolism were constructed using the neighbor-joining method from *S. viridis, Z. mays*, *O. sativa* and *A. thaliana* (Fig. S4). For the MEP pathway, SiDXS1/2/3, SiIDI1/2 and SiGGPPS7a/b/c were clustered together into one group with orthologous proteins in *Z. mays* and *O. sativa*. For the MVA pathway, SiAACT1/2/3 were more highly homologous proteins with ZmAACT1/2. SiMVD1/2 were higher homologous with OsMVD. SiHMGS1/2/3 were clustered into one group with ZmHMGS and OsHMGS. Moreover, SiHMGR1/2/3 were more highly homologous with ZmHMGR1 OsHMGR3-like and OsHMGR3. For the carotenoid biosynthesis and degradation pathway, SiPSY1/2/3, SiBCH1/2, SiZEP1/2, SiNCED1a/1b/4/5, and SiCCD7/8a/8b/8c were clustered into one group with orthologous proteins of *Z. mays* and *O. sativa*.

The promoters of these genes indicated that they were mainly involved with ‘light responsiveness’, ‘plant hormone’, ‘drought stress resistance’, ‘MYB binding site’, ‘endosperm specific’ and ‘seed specific’ cis-acting elements. Except for *SiMDC1* and *SiNCED1a,* the promoters of the remaining 52 genes contained a few ABA (abscisic acid) hormone cis-acting elements. Moreover, the promoters of all 54 genes had many MYB binding sites. Of them, the promoters of 51 and 14 genes had some ‘drought stress resistance’ and ‘light responsiveness’ cis-acting elements, respectively (Fig. S5).

### Differentially expressed genes during the panicle development stage

Eighteen samples, including two cultivars at three panicle development stages, were sequenced and analyzed by RNA-seq. These samples showed higher repeatability and dependency for the same panicle development stage. In total, 125.69 Gb raw read data were produced from 18 samples by RNA-seq, with Q30 ≥ 92.31%. The range of raw read numbers mapped to the reference genome among these samples was from 83.09 to 94.63%. By comparing the gene expression levels of DHD_S1 to that of DHD_S2 and DHD_S3, 2218, 3173 and 526 differentially expressed genes were identified, respectively. DHD had a total of 2218 genes with expression differences between S1 and S2 (D1), 526 genes with expression differences between S2 and S3 (D2), and 3173 genes with expression differences between S1 and S3 (D3). Similarly, JG21 had 2209 genes with expression differences between S1 and S2 (J1), 1544 genes between S2 and S3 (J2), and 3412 genes between S1 and S3 (J3). Among them, the number of DEGs between D1 and D2 was at least 24, and the number of DEGs between D1 and D3 was at most 1784. D2 vs. D3, J1 vs. J2, J2 vs. J3, and J1 vs. J3 had 244, 187, 848 and 1184 differentially expressed genes, respectively. The number of differentially expressed genes shared by the D1, D2, and D3 stages was 209, while that of the J1, J2, and J3 stages was 175.

DEGs between DHD and JG21 at different stages of ear development were analyzed (Fig. S6). There were 2690 DEGs between DHD and JG21 (C1) in the S1 period, 2149 DEGs between DHD and JG21 (C2) in the S2 period, and 604 DEGs between DHD and JG21 (C3) in the S3 period. In C1 vs. C2, C2 vs. C3, C2 vs. C3, there were 835, 66, and 61 genes were differentially expressed simultaneously and 227 genes were differentially expressed in all three periods.

The GO enrichment analysis showed that these DEGs mapped to “cellular processes”, “environmental information processing”, “genetic information processing”, “metabolism” and “organismal system”. The KEGG enrichment analysis showed that these DEGs mainly mapped to the ‘phenylpropanoid biosynthesis’, ‘flavone and flavanol biosynthesis’ and ‘flavonoid biosynthesis’ pathways between DHD and JG21 at the three panicle development stages (Fig. S7). However, in the S1 and S3 periods, the DEGs were mainly enriched in the ‘carotenoid biosynthesis’ pathway (Fig. S8).

### Gene expression characteristics related to carotenoid metabolism

To investigate the differential expression levels of genes related to the carotenoid metabolism pathway, we analyzed 54 gene expression values from the RNA-seq of JG21 and DHD at three panicle development stages (Fig. S9). In the MEP pathway, the *SiHDS* gene was more highly expressed at the S1 stage of DHD and JG21 than the other genes, with TPM values of 60.3 and 60.4, respectively. However, the *SiIDI3* gene had nearly no expression at the three panicle development stages of DHD and JG21. In the MVA pathway, the highest expression level was observed for the *SiHMGS3* gene during panicle development of DHD and JG21, with TPM values ranging from 41.0 to 70.5. However, *SiMVK* had the lowest expression level compared to other genes. In the carotenoid biosynthesis pathway, only *SiPDS* had a higher expression level, with TPM values ranging from 54.3 to 70.7. The remaining genes had both lower expression levels, with TPM values ranging from 0.0 to 25.9. Moreover, only *SiNCED1a* had a higher expression level than the other genes during the panicle development stage of the two cultivars. The others had lower expression levels for the two cultivars.

### Relationship between carotenoid metabolites and gene expression levels

Based on Pearson’s correlation coefficient analysis, the relationship between the expression levels of genes related to carotenoid metabolism and major carotenoid metabolite contents during the panicle development stage of the two cultivars was investigated (Fig. S10). *SiDXS3, SiMCT, SiHDR, SiIDI2, SiAACT1/3, SiMVK-like, SipMVK, SiPSY1, SiZDS and SiZEP1/2* were significantly positively correlated with the neoxanthin content at *P* < 0.05. However, *SiCCD8b* was significantly negatively correlated with the neoxanthin content at *P* < 0.05. Moreover, *SiDXS3, SiMCT, SiAACT3, SiZEP1/2*, and *SiCCD8a/8c/8d* were significantly positively correlated with the violaxanthin content at *P* < 0.05. *SiMCT, SiAACT3, SiPSY1, SiZEP2,* and *SiCc8a/8d* were significantly positively correlated with the lutein content at *P* < 0.05. Remarkably, *SiZEP1/2* was significantly positively correlated with four carotenoid metabolites except zeaxanthin (*r* = 0.882, 0.903, 0.974, 0.894, 0.899, 0.969, 0.914 and 0.909, respectively). However, *SiPSY2* and *SiNCED1b/4* were significantly positively correlated with the zeaxanthin content. *SiCMT, SiHDR, SiIDI2, SiAACT3, SiPSY1* and *SiZEP12* were significantly positively correlated with the β-carotene content. Moreover, *SiBCH1* was significantly negatively correlated with the β-carotene content (*r* = − 0.818, at *P* < 0.05). Furthermore, *SiPSY1* was significantly positively correlated with the contents of neoxanthin, lutein, and β-carotene at *P* < 0.05 (*r* = 0.921, 0.818 and 0.833, respectively). *SiCCD8b* was negatively correlated with all carotenoids except zeaxanthin (*P* < 0.05, *r* = − 0.903 and − 0.811; *P* > 0.05, *r* = − 0.792 and − 0.804).

### Carotenoid metabolism related to the gene coexpression network

**Fig. 5:**
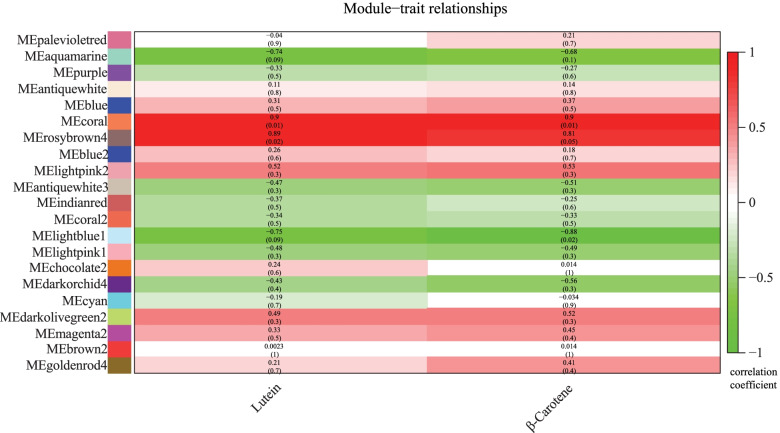
Correlation analysis of the carotenoid content in the gene coexpression network module

**Fig. 6:**
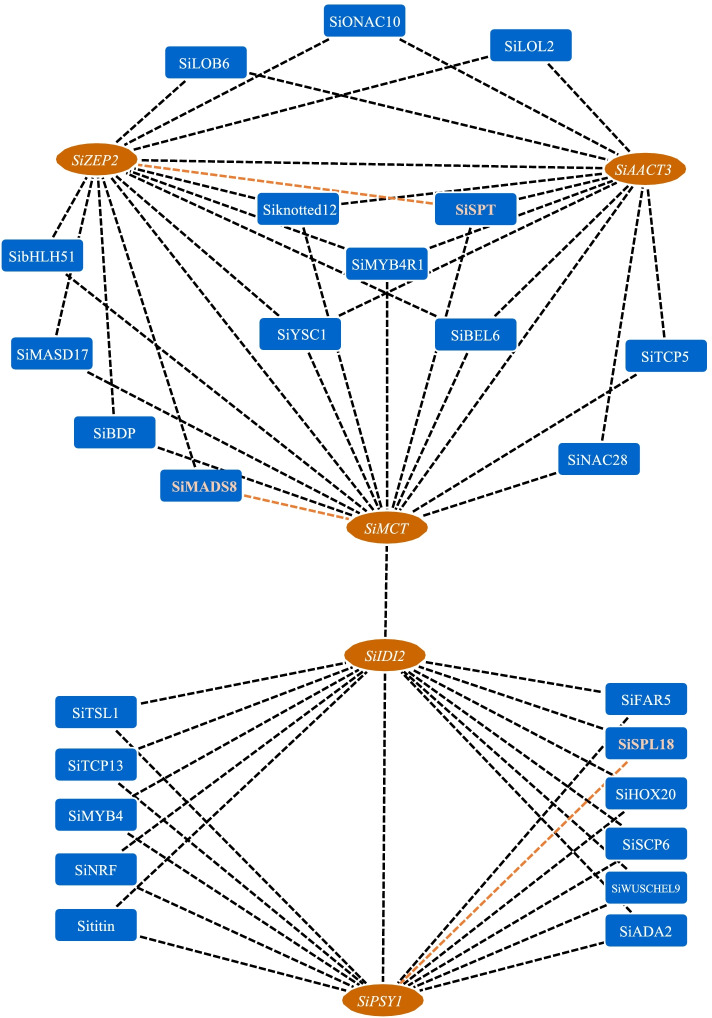
Coexpressionnetwork of important carotenoid metabolism genes andrelated transcriptionfactors. (The black dashed line represents the coexpressionof the two genes, and the brown dashed line represents the potential combination of the two genes predicted by FIMO)

The total gene expression value obtained from the RNA-seq data was used to construct a coexpression network. All genes were divided into 22 coexpression modules based on the WGCNA method (Fig. S11). The major carotenoid metabolites correlated with the coexpression network module were investigated and analyzed. Of the 22 modules, the MEcoral module was more highly correlated with lutein and β-carotene. Interestingly, the module contained 13 key genes involved in the carotenoid metabolism pathway, which were composed of *SiMCT, SiIDI2, SiGGPPS7a, SipMVK-like, SipMVK, SiAACT1/3, SiPDS, SiPSY1, SiBCH1, SiZEP1/2* and *SiCD8b* (Fig. 5 and Table S2). Based on the previous analysis of the correlation between carotenoid metabolism genes and carotenoid content (Fig. S10), we selected 5 genes which were respectively correlated with Lutein and β-carotene to construct a coexpression network. In this coexpression network, we found that 25 transcription factors had higher weight values related to *SiIDI2/SiMCT/SiAACT3/SiPSY1/SiZEP2* (Tables S3, S4 and Fig. 6). These transcription factors showed down-regulated expression patterns at different stages of DHD and JG21 panicle developments. Of all transcription factors, *SiMADS8* had the highest expression level was at the S1 stage of DHD. The *SiWUSCHEL9/SibHLH51/SiNAC28* had lower expression levels at all developmental stages of the two foxtail millet varieties (Fig. S12). Finally, based on analysis result of FIMO, the known binding motifs of *SiMADS8/SiSPL18/SiSPT* were CACATTTTTGT, GGTACGGT and ACCACGTGT located on promoters of *SiMCT/SiPSY1/SiZEP2* respectively (Table S5). Specifically, the regulated *SPL* gene could be a conserved repressor to regulate *PSY* and carotenoid metabolism flux according to a previously reported reference. These results suggested that the gene coexpression network related to the carotenoid metabolism pathway was available and receivable.

### qRT-PCR verified the expression of key genes related to carotenoid metabolism

The relative expression levels of key genes related to carotenoid metabolism during the panicle development stage of DHD were determined by qRT-PCR. We found that SiDXS3, SiPSY1/2 and SiGGPP7a /c, as major rate-limiting enzymes during the panicle developmental stages, showed a consistent downregulation trend. But only the expression of *SiPY2* and *SiGGPP7c* were significantly decreased Among them, the expression levels of *SiDXS1, SiPSY1* and *SiGGPP7a/c* were 1.6-, 2.04-, 2.35- and 4.91-times higher at the S1 stage compared with the S3 stage, respectively. The expression levels of *SiDXS1*, *SiPSY2/3* and *SiGGPP7b* in JG21 cells were higher than those in DHD cells at the S1 stage. The *SiDXS1* expression level in JG21 was 9.58-times higher than that in DHD at the S1 stage (Fig. S13–1).

For the above coexpression gene pairs, the expression levels of *SiIDI2, SiZEP2, SiMCT, SiSPL18* and *SiMADS8* sharply declined during the panicle development stage. The expression level of *SiSPL18* was 3.55-times higher at the S1 stage than at the S3 stage in DHD. The expression level of *SiMADS8* in DHD was 1.97-times higher than that of JG21 at the S1 stage. The expression levels of *SiZEP2, SiSPL18* and *SiMADS8* at the S1 stage in DHD were 1.12-, 1.15- and 1.97-times higher than those in JG21. Moreover, *SiIDI2* expression was 1.23-fold higher in JG21 cells than in DHD cells. Based on the correlation analysis, there was a stronger correlation between the relative expression value and TPM value, with *R*^2^ = 0.9982 (Fig. S13–2). This finding suggests that these analysis results were reliable.

## Discussion

### Characteristics of carotenoid content variations in plants

With an increasing number of people on our planet, food security issues and the energy crisis are increasingly outstanding and currently face challenges for developing areas [33]. Specifically, many people have presented worsening health statuses because of a lack of dietary vitamin intake for the human body. Carotenoids, as essential vitamins, cannot be synthesized by humans and animals, which play an important role in antioxidation of reactive oxygen and reducing the risk of modern civilization diseases, i.e., cancer, cardiovascular or photosensitivity disorders [12]. To date, more than 750 different carotenoid metabolites have been detected and annotated from the natural world from bacteria, algae and higher plants [34–36]. Although golden rice can help effectively alleviate vitamin A deficiency via the food supply for people living in developing areas, natural food enriched in vitamin A is more easily accepted than GMF (genetically modified food). Previously, reports showed that carotenoid metabolites were enriched in the grains of foxtail millet, although the dynamic changes in the carotenoid content and species are not well understood at present. For Arabidopsis leaves, the carotenoid metabolites are mainly composed of lutein, β-carotene, neoxanthin and violaxanthin. Lutein is a major carotenoid metabolite that accounts for 46.55% of the total carotenoid content [36]. In corn, lutein and zeaxanthin are the most abundant carotenoid metabolites in all immature and mature grains [37]. Among 201 corn inbred lines with different grain colors, significant differences in carotenoid content occurred, with the total carotenoid content ranging from 95.5 to 629.6 μg /100 mg. Moreover, the most abundant carotenoids in corn kernels were zeaxanthin (14.43 μg /100 mg) and lutein (12.32 μg /100 mg) [38]. In wheat and wheat bread, lutein is the main carotenoid component (0.72–3.07 μg /100 mg), followed by zeaxanthin (0.88–1.84 μg /100 mg) and β-carotene (0.07–0.33 μg /100 mg) [31, 32]. The main carotenoid metabolites in brown rice are β-carotene and lutein, up to 1.50 μg /100 mg and 1.09 μg /100 mg, respectively, while the content of zeaxanthin was as low as 0.37 μg /100 mg [14]. The total carotenoid content in mature grains of foxtail millet (192.3 μg /100 mg) was 10- to 100-times higher than that in the above cereal crops [11]. In our study, the major carotenoid metabolites were lutein (47.35%) and β-carotene (44.62%) (Fig. S3). Moreover, we found a significant difference in the total carotenoid content in the grains of foxtail millet between the two varieties at *P* < 0.05. A comparison between DHD and JG21, which have white color grains and yellow color grains, respectively, at the early stage of grain showed that the content of lutein and β-carotene in DHD (11.51 μg /100 mg and 12.56 μg /100 mg, respectively) was significantly higher than that in JG21 (8.94 μg /100 mg and 9.86 μg /100 mg, respectively). However, at the end of development, the contents of the two types of carotene in the white-grained variety DHD were 5.43 μg /100 mg and 2.59 μg /100 mg, respectively, which were lower than that of the yellow-grained variety JG21 (5.61 μg /100 mg and 4.21 μg /100 mg, respectively) during this period (Fig. 1).

### Key genes related to carotenoid metabolism controlled carotenoid metabolism flux

A total of 54 genes related to carotenoid metabolism were identified and analyzed in foxtail millet. Furthermore, we found that these genes had stronger genomic collinearity relationships among cereal crops. We also found that the genome distribution characteristics of these genes were similar to the results of *Brassica napus* [39], indicating that they were essential for maintaining plant development, physiology and biochemical processes in plants.

Carotenoid precursor metabolites are formed by the condensation of the 5-carbon precursors IPP and DMAPP, which are produced through the MEP pathway in plastids [40]. DXS and DXR, as important rate-limiting enzymes in the MEP pathway, play an important role in the regulation of carotenoid metabolism flux [41]. For foxtail millet, the results of the phylogenetic tree showed that the three *SiDXS* genes were divided into three independent branches, suggesting that each *SiDXS* gene could play a different role in the biosynthesis of terpenoids. Similar results existed in other plants [42–44]. Moreover, our results showed that *SiDXS3* was specifically expressed in the panicle of foxtail millet, which indicated that this gene could be the key gene for the carotenoid accumulation at the early panicle development stage. The *IDI* gene was responsible for regulating the ratio of IPP and DMAPP products in the MEP and MVA pathways [45]. Overexpression of different exogenous *IDI* genes in *E. coli* could promote the biosynthesis of β-carotene, lycopene, astaxanthin and zeaxanthin in vivo [46–48]. Our results showed that the *IDI2* gene was more highly expressed at the panicle development stage among the three *IDI* gene members correlated with the content of major carotenoid metabolites.

The cytoplasmic MVA pathway also contributes to the synthesis of IPP and DMAPP, which provide precursors for the biosynthesis of sesquiterpenes, polyterpenes, sterols, and glycols and the formation of ubiquinone in mitochondria. In Arabidopsis, the expression level of *AtAACT2* was six times that of *AtAACT1* [49]. In our study, the expression of *SiAACT1* was also significantly higher than that of *SiAACT2/3*, which indicated that *SiAACT1* plays a more important role in cytoplasmic isoprenoid biosynthesis during the panicle development stage of foxtail millet. PSY, as a rate-limiting enzyme in the carotenoid biosynthesis pathway, is easily regarded as the main bottleneck of carotenoid circulation. Ectopic expression of the *PSY* gene and *PaCRTI* gene in rice created the first- and second-generation ‘golden rice’, which had higher carotenoid contents of 16 μg /100 mg and 367 μg /100 mg, respectively [22, 50]. Moreover, overexpression of *PSY1* in tomato plants significantly increased the carotenoid content in tomato fruit [51, 52]. Our results showed that the three *PSY* genes have differentially expressed patterns during the panicle development stage, suggesting that they have differential functions in regulating carotenoid metabolism flux [53]. Additionally, many studies have demonstrated that *ZEP* is an important node for fine-tuning carotenoid metabolism in Arabidopsis [54, 55]. The SNP variants of *ZEP* in sorghum and Arabidopsis were significantly correlated with the zeaxanthin content and lutein/zeaxanthin ratio [56, 57]. Meanwhile, we found that two members of the *ZEP* gene in foxtail millet were highly correlated with the content of carotenoid metabolites. However, the expression of *SiZEP2* was 72-times higher than that of *SiZEP1*, suggesting that *SiZEP2* could be a major gene for regulating carotenoid metabolism.

The function of CCDs as nonhaem iron-dependent enzymes was to cleave carotenoids by catalyzing the oxidative cleavage of a double bond to form either a ketone or an aldehyde, which reduced the carotenoid content in *Arabidopsis thaliana*, *Chrysanthemum morifolium*, *Fragaria × ananassa* and *Solanum tuberosum* [58–62]. In foxtail millet, *SiCCD8a, SiCCD8b* and *SiCCD8d* were tandem repeats distributed on chromosome 5. However, their expression patterns were significantly different. Moreover, we found that *SiCCD8a* and *SiCCD8d* were positively correlated with carotenoid metabolites while *SiCCD8b* was negatively correlated with them. Taken together, these results indicate that the molecular mechanism underlying carotenoid metabolism that maintains the balance of carotenoid synthesis and degradation during the panicle development stage in foxtail millet is relatively complex and challenging.

### Transcription regulation network related to carotenoid metabolism

In recent years, a number of studies have shown that some transcription factors directly or indirectly regulate the expression of carotenoid metabolism and metabolism genes and further control carotenoid metabolism flux in plants. To date, research reports on the interactive relationship between some transcription factors and genes related to carotenoid metabolism have been identified and proven in *Arabidopsis*. Nevertheless, the regulatory network related to the carotenoid metabolism pathway is still unknown and lacks in-depth investigation. In our coexpression network, we found that 25 transcription factors had a stronger interaction with five genes related to carotenoid metabolism. However, only the interaction relationship between SPT and *ZEP2,* SPL18 and *PSY1,* MADS8 and *MCT* could be identified and predicted through the hTFtarget tool. A well-known gene named PHYTOCHROME INTERACTING FACTOR 1 (PIF1), which is a key transcription regulator of carotenoid biosynthesis, could be activated by phytochrome under red light signal treatment during the process of seedling deyellowing [63]. PIF1 is phosphorylated by phytochrome when activated by light and subsequently degraded by the proteasome by inhibiting the expression of *AtPSY* in the dark [19, 64]. PIF1 also initiates genes related to chlorophyll biosynthesis and chloroplast development [65]. Another important bZIP transcription factor, LONG HYPOCOTYL 5 (HY5), could antagonize PIF1 during the process of photomorphogenesis [20, 66]. Spatula (SPT) annotated a bHLH transcription factor that regulates ABA metabolism to control the gynoecium and promote vegetative growth and seed dormancy [67–70]. Hence, we speculated that SPT, as a key regulator of carotenoid metabolism to ABA, had a stronger interaction relationship with *SiZEP2* in the panicle of foxtail millet [71].

In tomato, an SPL gene mutant with a colorless and immature tomato could not produce lycopene because *PSY1* is not expressed [72]. Overexpression of *AtmiR156b*, which repressed *AtSPL3* expression [73], enhanced the content of lutein and β-carotene in rape seeds [74, 75]. Hence, we suggest that the *SiPSY1* gene interacts with the *SPL18* gene as a potential regulator of carotenoid metabolism.

Several MADS-box regulators affect the expression of the tomato *CBP* gene, including tomato AGAMOUS-like 1 (TAGL1), RIPENING INHIBITOR (RIN), and FRUITFULL1/2 (FUL1/2) [76]. These MADS-box proteins directly or indirectly positively regulated the expression of *SlPSY1, SlPSY2, SlZDS, SlZ-ISO, SlCRTISO and SlBCH* while negatively regulating the expression of *SlLCYB* and *SlLCYE* [77–82]. Our results showed that the *SiMADS8* and* SiMCT* genes had a stronger coexpression relationship.

Based on these results, we obtained a gene coexpression network related to carotenoid metabolism and found that three gene pairs had a stronger interaction relationship. These results would lay the foundation for exploring the underlying molecular regulation mechanism of carotenoid metabolism in the panicle of foxtail millet.

## Supplementary Information

**Additional file 1.**

## Data Availability

All datasets supporting the results of this article are included within the article and its supplementary information.
