# Supplementary material for: Comparative metabolomic and transcriptomic analysis reveals a coexpression network of the carotenoid metabolism pathway in the panicle of Setaria italica
Source: BMC Plant Biol. 2022 Mar 8;22:105. doi: 10.1186/s12870-022-03467-2 (PMC8903627; doi:10.1186/s12870-022-03467-2)
Supplement: Supplementary file 1 — Additional file 1. [file 12870_2022_3467_MOESM1_ESM.pdf]

Table S1. qRT–PCR primer sequence information.

Gene		Sequence (5'->3')	Tm		Sequence (5'->3')	Tm
SiDXS1	Forward primer	CTTCCACGTCTCCAAGACCG	60.39	Reverse primer	ATCTTGTGCGGGTACGACTG	60.11
SiDXS2	Forward primer	ATCTAAGTCCCCAACGCTGC	60.11	Reverse primer	TTCTGGAAGTGGTTGAGCCC	59.89
SiDXS3	Forward primer	ATTCTCACAGGAAGGCGCTC	60.11	Reverse primer	CAAGGCCAGCGGAAAGACTA	60.04
SiGGPPS7a	Forward primer	TGGCAAGTGACAAGACGACA	59.82	Reverse primer	TGCAATAGAGGTGCTGCCTT	59.67
SiGGPPS7b	Forward primer	ACGCTCCTCAGAGATCCACT	60.03	Reverse primer	GGTGACTTTGGGGGCTGTAA	59.89
SiGGPPS7c	Forward primer	GTCCGGGAACGGTAAGATGA	59.18	Reverse primer	GTACTTGTCCCCGAACCTGT	59.32
SiPSY1	Forward primer	CTACATTACGCCGACAGCCT	59.9	Reverse primer	AGCGGCATCAAGCATATCGT	60.25
SiPSY2	Forward primer	TCTACCTCCCACAGGACGAG	60.03	Reverse primer	GTGATCTGGTCCCGCATGAA	60.11
SiPSY3	Forward primer	ATGACTCCTGAAAGGCGCAA	59.96	Reverse primer	CGATCAAGAGCAGTCGGTGT	60.11
SiMCT	Forward primer	CTTGGTGTGCCTGTGAAAGC	59.97	Reverse primer	AGAGTTTTCCGGTCAAGGGT	58.86
SiIDI2	Forward primer	GGAAGGGGAAAAACCTGGCT	60.18	Reverse primer	GTCCAGCGGGGGACATAAAT	59.82
SiZEP2	Forward primer	TGGGATCAGGTGATTGGCTC	59.45	Reverse primer	ATTGGGTTGGGGTTGTGGAG	60.18
SiSPL12	Forward primer	GCTTATTTTCGAGTGCAGGGAC	59.9	Reverse primer	CTTGATGACCCCAGACCAGC	60.39
SiMADS8	Forward primer	GAACAAGATCAACCGCCAGG	59.2	Reverse primer	AAGTGTTTTCGTGATGCTTTGC	58.88

Table S2. Carotenoid metabolism genes in highly correlated coexpression modules.

Modules	Gene ID	Gene names
coral	Si2g34540	SiIDI2
coral	Si2g36780	SiGGPPS7a
coral	Si3g26580	SiMVK
coral	Si4g27520	SiPSY1
coral	Si5g31350	SiAACT3
coral	Si5g32040	SiCCD8b
coral	Si5g40650	SiMCT
coral	Si7g13100	SiZEP2
coral	Si7g13140	SiZEP1
coral	Si7g28520	SiAACT1
coral	Si9g45700	SipMVKp
coral	Si9g50120	SiPDS
coral	Si9g54300	SiBCH1
rosybrown4	Si5g32020	SiCCD8c
rosybrown4	Si5g32030	SiCCD8d
rosybrown4	Si8g10360	SiCCD8a

Table S3. Coexpression network interaction gene weight.

Carotenoid metabolism gene	Weight	Coexpressed transcription factors
SiMCT	0.386251394	SiSPT
SiAACT3	0.304878152	SiSPT
SiZEP2	0.361754272	SiSPT
SiMCT	0.384335618	SiMYB4R1
SiAACT3	0.271241661	SiMYB4R1
SiZEP2	0.300990223	SiMYB4R1
SiIDI2	0.385681291	SiNRF
SiPSY1	0.415965475	SiNRF
SiMCT	0.384821482	SiYSC1
SiAACT3	0.327917932	SiYSC1
SiZEP2	0.295975224	SiYSC1
SiIDI2	0.334503931	SiSPL18
SiPSY1	0.384859196	SiSPL18
SiMCT	0.378361038	SiMADS8
SiZEP2	0.285384126	SiMADS8
SiAACT3	0.267816736	SiONAC10
SiZEP2	0.276874915	SiONAC10
SiMCT	0.392211687	SiBDP
SiZEP2	0.28761981	SiBDP
SiIDI2	0.387047815	SiTCP13
SiPSY1	0.403365169	SiTCP13
SiAACT3	0.309721273	SiLOL2
SiZEP2	0.279519029	SiLOL2
SiMCT	0.374868829	SiBEL6
SiAACT3	0.30455245	SiBEL6
SiZEP2	0.363999659	SiBEL6
SiIDI2	0.360921821	SiFAR5
SiPSY1	0.377958313	SiFAR5
SiMCT	0.416284646	SiTCP5
SiAACT3	0.279522109	SiTCP5
SiIDI2	0.36704721	SiWUSCHEL9
SiPSY1	0.413559103	SiWUSCHEL9
SiAACT3	0.280641888	SiLOB6
SiZEP2	0.275204488	SiLOB6
SiIDI2	0.372214437	SiHOX20
SiPSY1	0.403521752	SiHOX20
SiIDI2	0.34902706	SiMYB4
SiPSY1	0.403797375	SiMYB4
SiMCT	0.364150396	SiMASD17
SiZEP2	0.29251368	SiMASD17
SiMCT	0.381796069	SibHLH51
SiZEP2	0.280301113	SibHLH51
SiMCT	0.374419757	Siknotted12
SiAACT3	0.325483851	Siknotted12
SiZEP2	0.34030867	Siknotted12
SiIDI2	0.338332756	SiADA2
SiPSY1	0.383682891	SiADA2
SiIDI2	0.360921994	Sititin
SiPSY1	0.396113279	Sititin
SiIDI2	0.334971173	SiTSL1
SiPSY1	0.393624355	SiTSL1
SiMCT	0.360123208	SiNAC28
SiAACT3	0.284596672	SiNAC28
SiIDI2	0.358395972	SiSCP6
SiPSY1	0.38510165	SiSCP6

Table S4. List of transcription factors coexpressed with carotenoid metabolism genes, annotation information and interaction sites.

Gene ID	Motif ID	Name	Describtion
Si1g35900	MA1061.1	SiSPT	PREDICTED: transcription factor SPATULA-like [Setaria italica]
Si2g03800	-	SiMYB4R1	PREDICTED: myb domain protein 4r1 [Arabidopsis thaliana]
Si2g14340	-	SiNRF	PREDICTED: NAD(P)-binding Rossmann-fold superfamily protein [Arabidopsis thaliana]
Si2g25310	-	SiYSC1	PREDICTED: nuclear transcription factor Y subunit C-1-like [Setaria italica]
Si2g27120	MA1057.1	SiSPL18	PREDICTED: squamosa promoter-binding-like protein 18-like isoform X1 [Setaria italica]
Si2g27130	MA0563.1	SiMADS8	PREDICTED: MADS-box transcription factor 8-like isoform X1 [Setaria italica]
Si2g27330	-	SiONAC10	PREDICTED: NAC transcription factor ONAC010-like [Setaria italica]
Si2g42870	-	SiBDP	PREDICTED: B3 domain-containing protein Os07g0679700-like isoform X3 [Setaria italica]
Si3g17990	MA1067.1	SiTCP13	PREDICTED: transcription factor TCP13-like isoform X1 [Setaria italica]
Si3g37770	-	SiLOL2	PREDICTED: protein LOL2-like isoform X5 [Setaria italica]
Si3g39610	-	SiBEL6	PREDICTED: BEL1-like homeodomain protein 6-like isoform X1 [Setaria italica]
Si4g21630	-	SiFAR5	PREDICTED: protein FAR1-RELATED SEQUENCE 5-like isoform X1 [Setaria italica]
Si5g33210	MA1067.1	SiTCP5	PREDICTED: transcription factor PCF5-like [Setaria italica]
Si5g38630	-	SiWUSCHEL9	PREDICTED: WUSCHEL-related homeobox 9-like [Setaria italica]
Si5g40810	-	SiLOB6	PREDICTED: LOB domain-containing protein 6-like [Setaria italica]
Si6g18880	MA0951.1	SiHOX20	PREDICTED: homeobox-leucine zipper protein HOX20-like [Setaria italica]
Si7g14000	-	SiMYB4	PREDICTED: myb-related protein Myb4-like [Setaria italica]
Si7g22110	MA0584.1	SiMASD17	PREDICTED: MADS-box transcription factor 17-like isoform X1 [Setaria italica]
Si7g27870	MA0584.1	SibHLH51	PREDICTED: transcription factor bHLH51-like [Setaria italica]
Si9g07800	-	Siknotted12	PREDICTED: homeobox protein knotted-1-like 12-like [Setaria italica]
Si9g09290	-	SiADA2	PREDICTED: transcriptional adapter ADA2-like [Setaria italica]
Si9g13270	-	Sititin	PREDICTED: titin-like [Setaria italica]
Si9g46280	-	SiTSL1	PREDICTED: TSL-kinase interacting protein 1-like [Setaria italica]
Si9g54680	MA1046.1	SiNAC28	PREDICTED: NAC domain containing protein 28[Arabidopsis thaliana]
Si9g56010	-	SiSCP6	PREDICTED: scarecrow-like protein 6-like [Setaria italica]

Table S5. cis-acting elements of carotenoid metabolism genes predicted that can bond with coexpressed transcription factors in MEME.

Motif ID	Alt ID	Sequence Name	Strand	Start	End	p-value	q-value	Matched Sequence
MA0563.1	SiMADS8	SiMCT	+	639	649	0.0000913	0.723	CACATTTTTGT
MA1057.1	SiSPL18	SiPSY1	-	1617	1624	0.0000901	0.718	GGTACGGT
MA1061.1	SiSPT	SiZPE2	-	1074	1082	0.0000412	0.492	ACCACGTGT

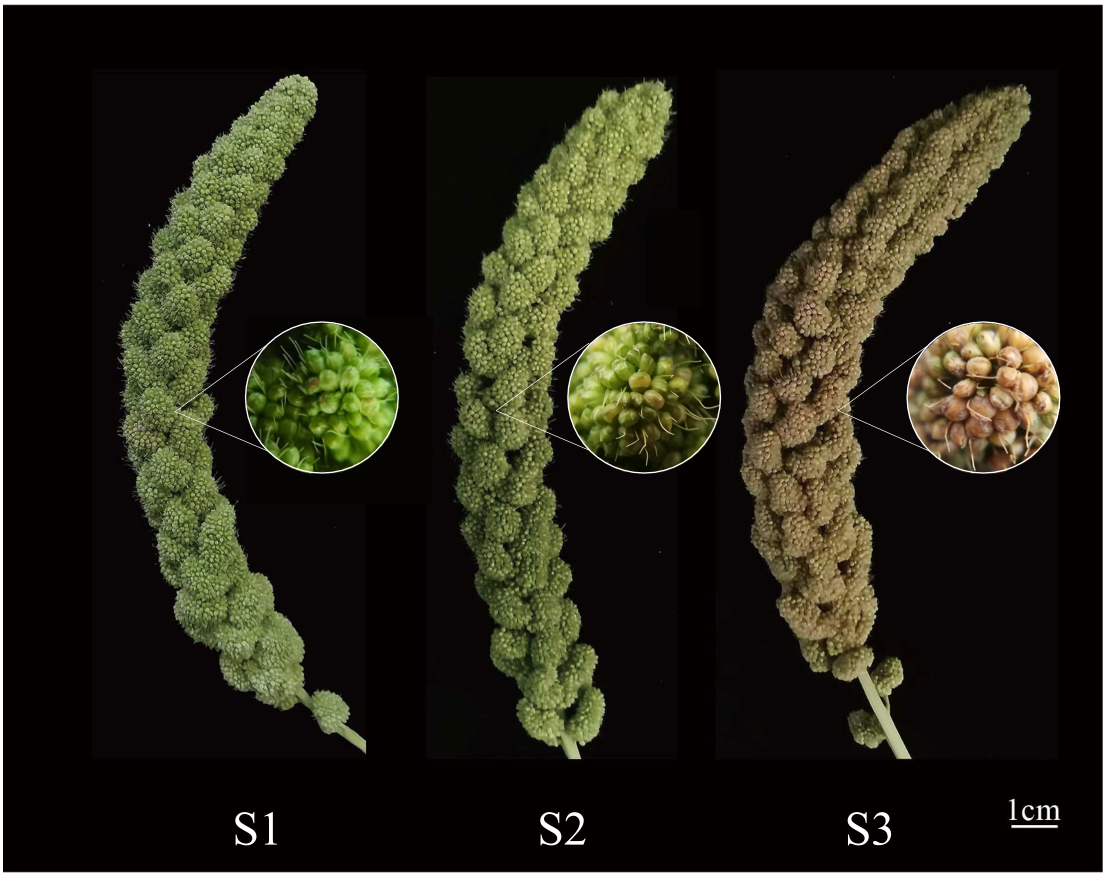

Figure S1. Phenotypic differences in the panicle at the early, middle and end stages of development. (S1: Beginning of diaspore colouring, 111 days after imbibition, S2: Colouring of half of diaspores, 129 days after imbibition and S3: Colouring of almost all diaspores, 143 days after imbibition)

A

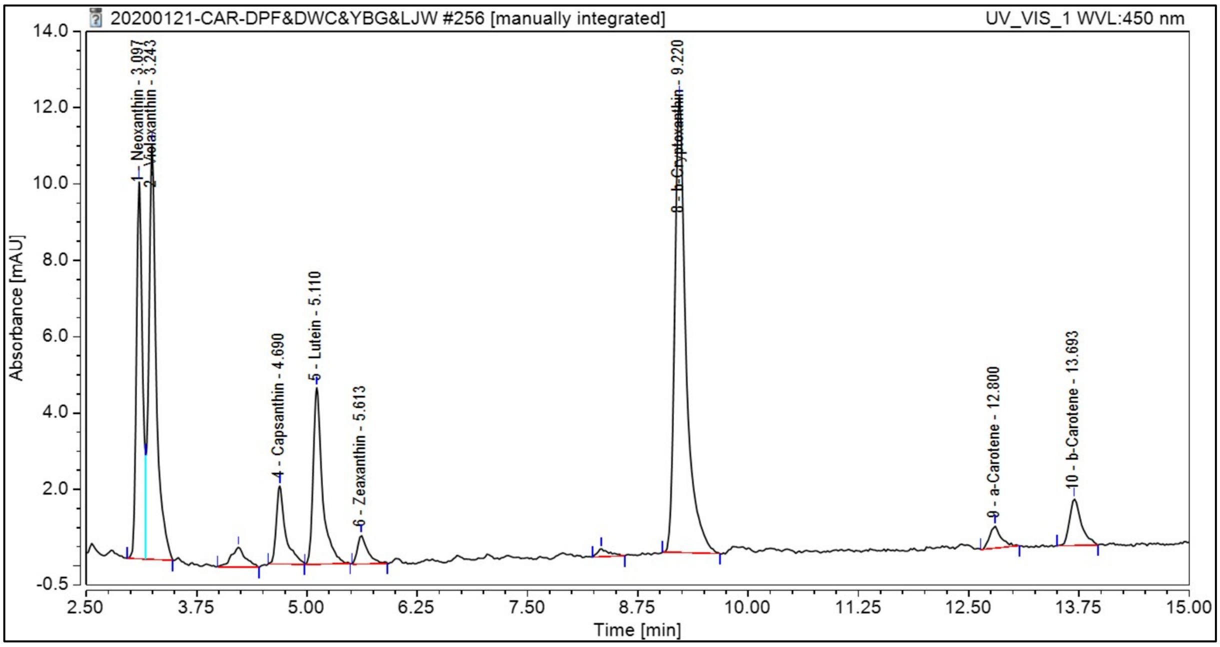

B

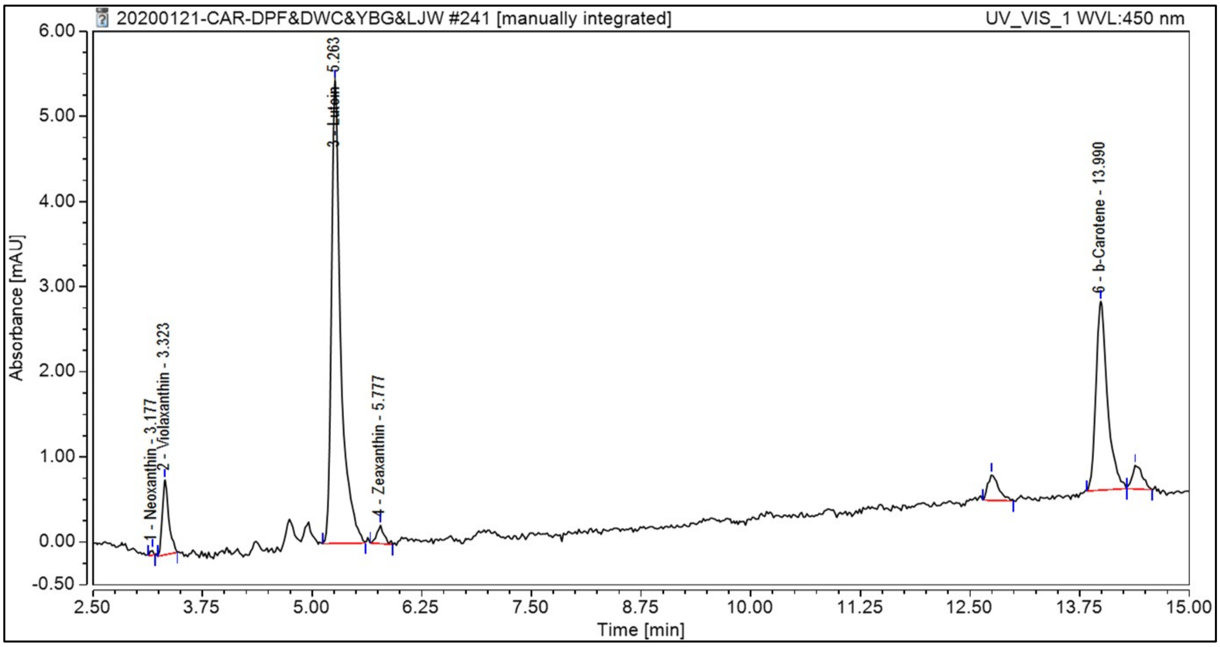

Figure S*2*. Chromatogram of identified carotenoids. (A) Chromatogram of the standard product. (B) Chromatogram of the sample.

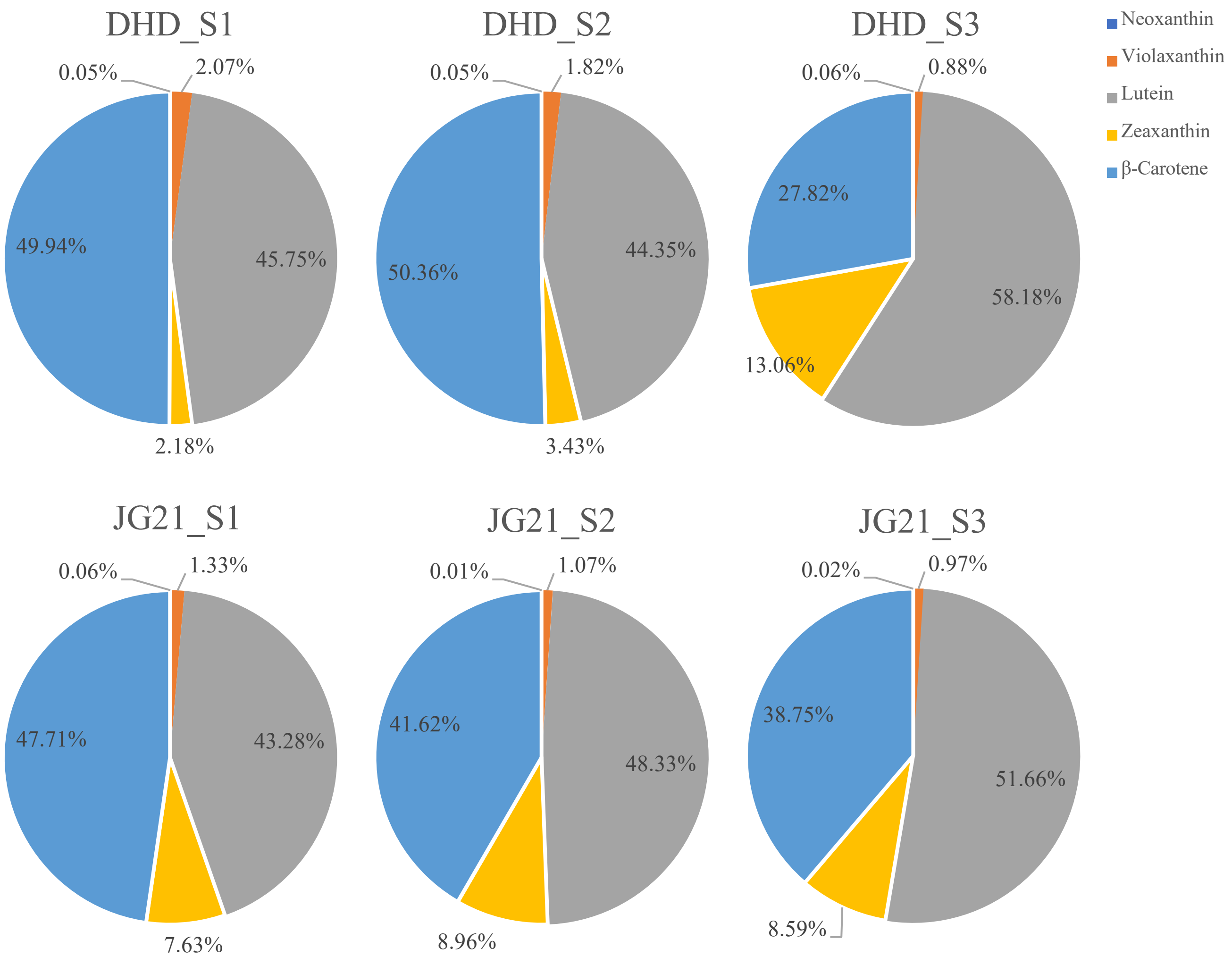

Figure S3. Composition of carotenoids in DHD and JG21 during panicle development at the early (S1), middle (S2) and mature (S3) stages.

A

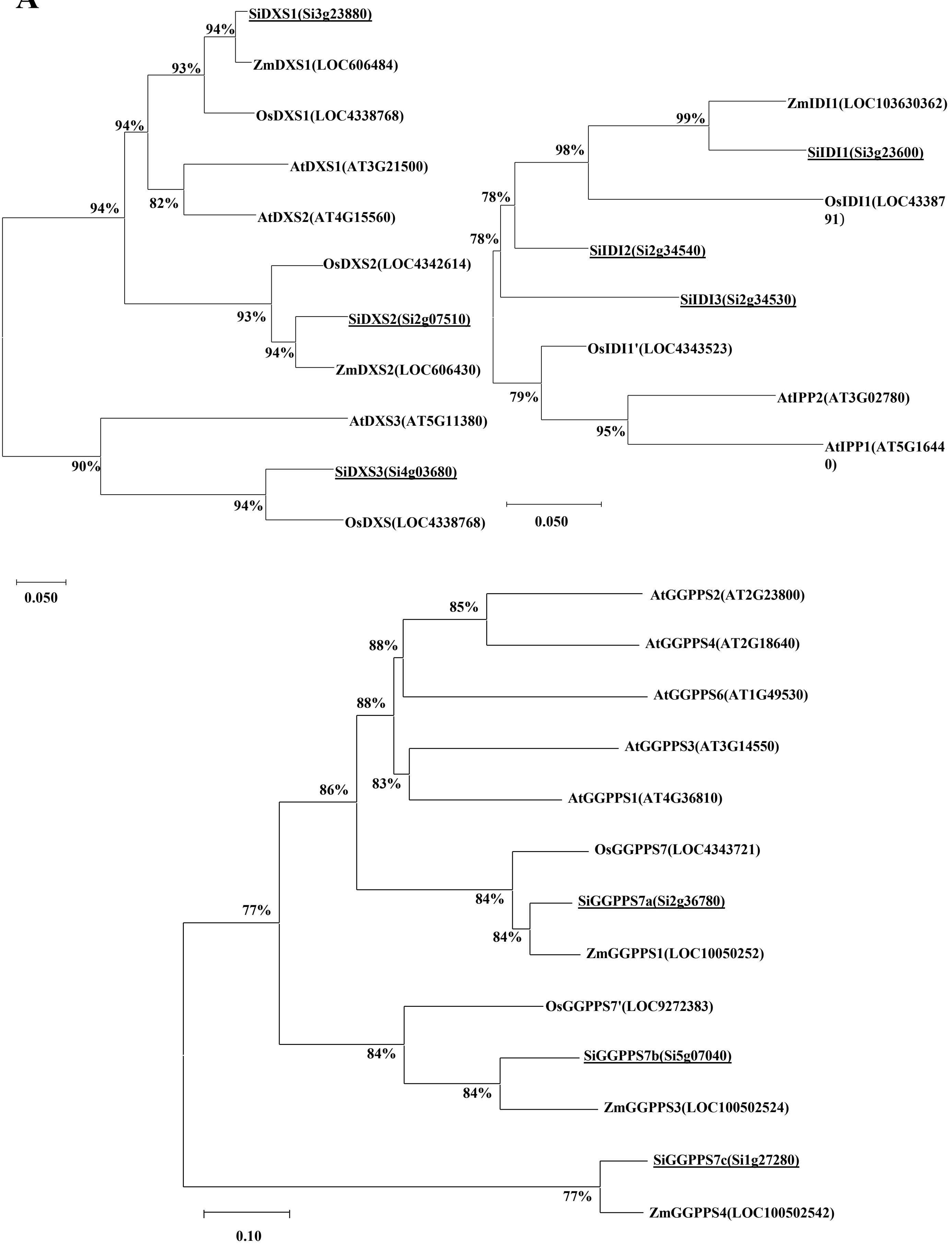

Figure S4. Genetic evolution analysis of the carotenoid metabolism pathway gene family members: (A) MEP pathway, (B) MVA pathway, (C) carotenoid biosynthetic pathway, and (D) carotenoid degradation pathway.

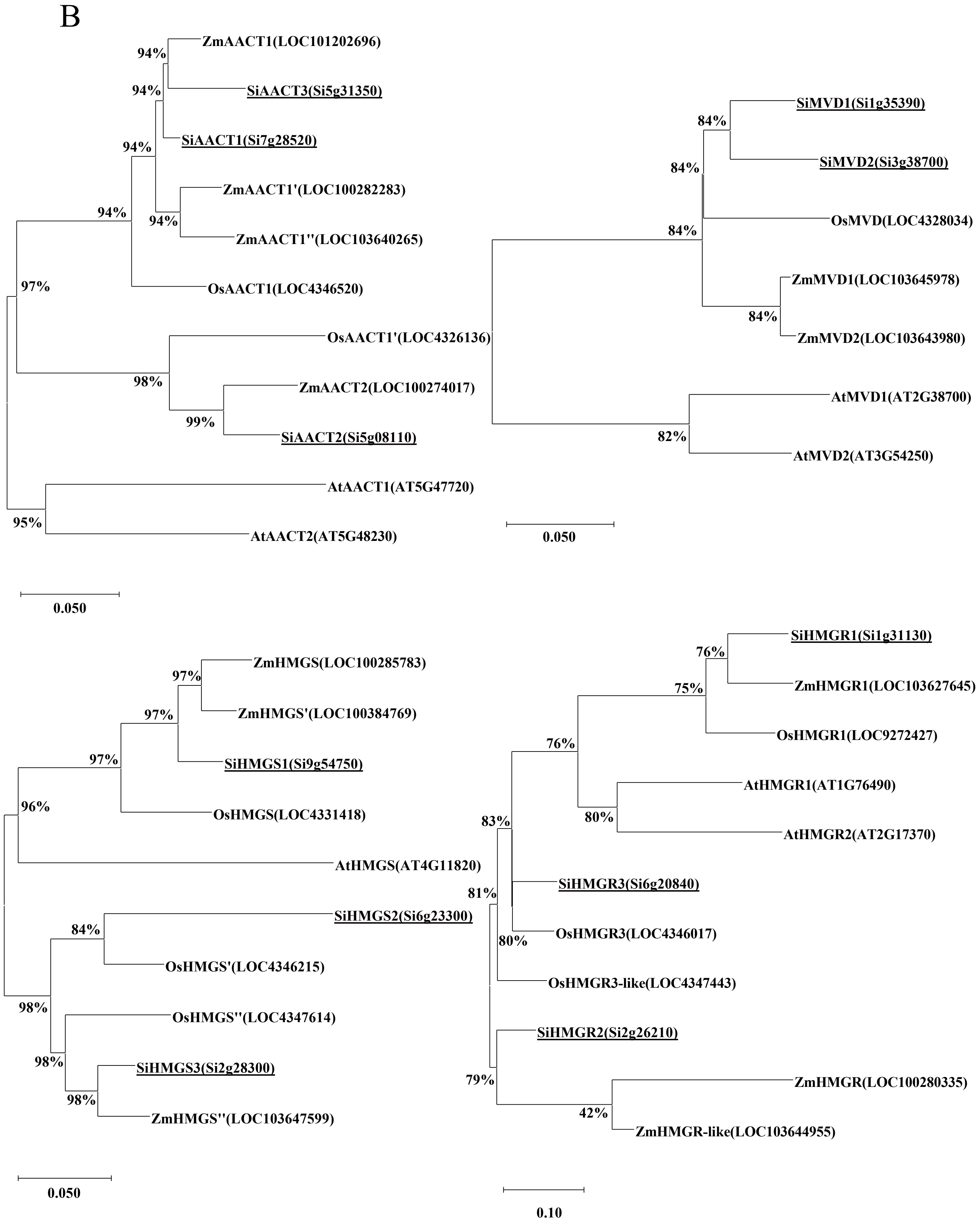
B
ZmAACT1(LOC101202696)
SiAACT3(Si5g31350)
SiAACT1(Si7g28520)
ZmAACT1'(LOC100282283)
ZmAACT1''(LOC103640265)
OsAACT1(LOC4346520)
OsAACT1'(LOC4326136)
ZmAACT2(LOC100274017)
SiAACT2(Si5g08110)
AtAACT1(AT5G47720)
AtAACT2(AT5G48230)
94%
94%
94%
94%
94%
97%
98%
99%
95%
0.050
SiMVD1(Si1g35390)
SiMVD2(Si3g38700)
OsMVD(LOC4328034)
ZmMVD1(LOC103645978)
ZmMVD2(LOC103643980)
AtMVD1(AT2G38700)
AtMVD2(AT3G54250)
84%
84%
84%
84%
82%
0.050
ZmHMGS(LOC100285783)
ZmHMGS'(LOC100384769)
SiHMGS1(Si9g54750)
OsHMGS(LOC4331418)
AtHMGS(AT4G11820)
SiHMGS2(Si6g23300)
OsHMGS'(LOC4346215)
OsHMGS''(LOC4347614)
SiHMGS3(Si2g28300)
ZmHMGS''(LOC103647599)
97%
97%
97%
96%
84%
98%
98%
98%
0.050
SiHMGR1(Si1g31130)
ZmHMGR1(LOC103627645)
OsHMGR1(LOC9272427)
AtHMGR1(AT1G76490)
AtHMGR2(AT2G17370)
SiHMGR3(Si6g20840)
OsHMGR3(LOC4346017)
OsHMGR3-like(LOC4347443)
SiHMGR2(Si2g26210)
ZmHMGR(LOC100280335)
ZmHMGR-like(LOC103644955)
76%
75%
76%
80%
83%
81%
80%
79%
42%
0.10

C

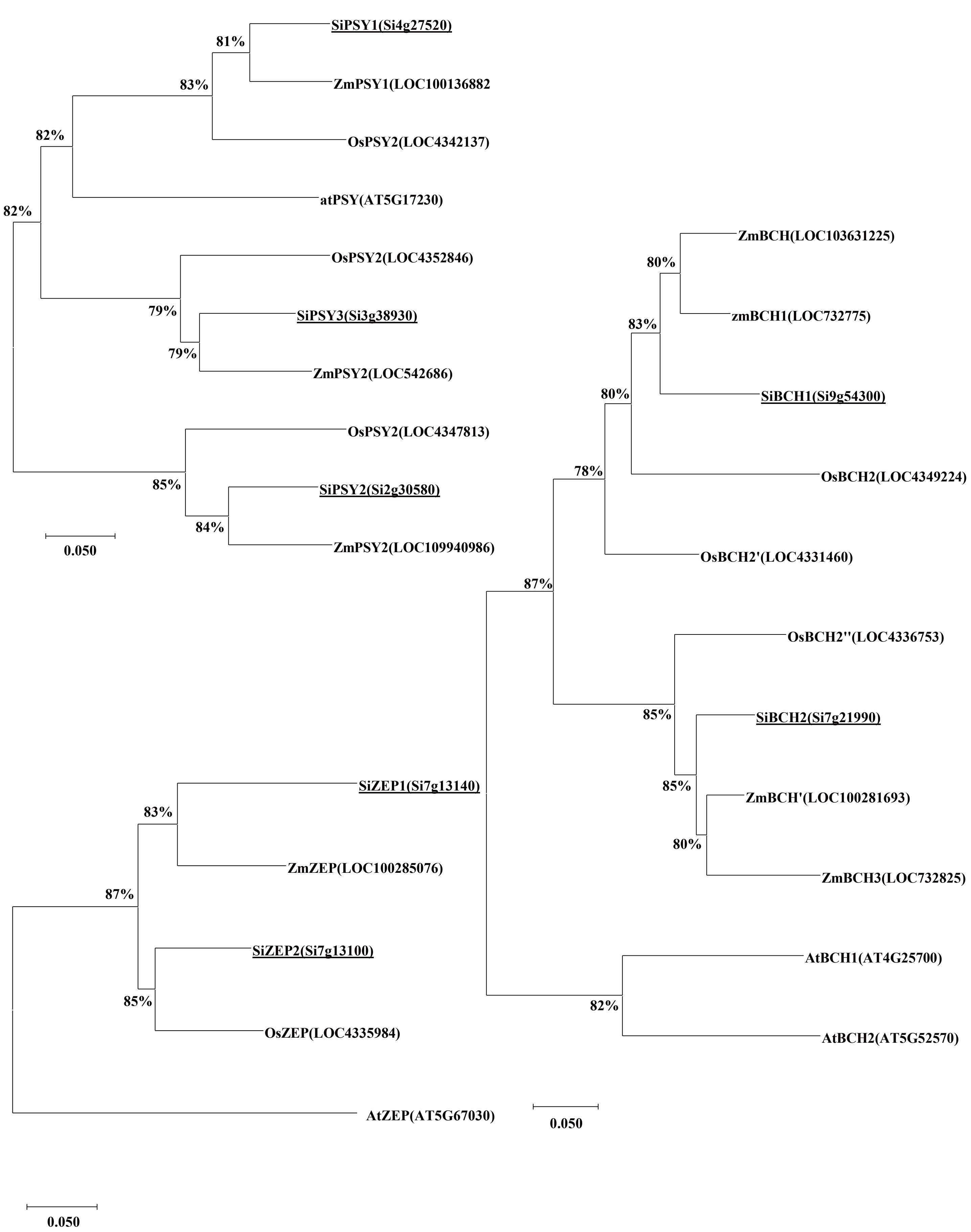

SiPSY1(Si4g27520)
ZmPSY1(LOC100136882
OsPSY2(LOC4342137)
atPSY(AT5G17230)
OsPSY2(LOC4352846)
SiPSY3(Si3g38930)
ZmPSY2(LOC542686)
OsPSY2(LOC4347813)
SiPSY2(Si2g30580)
ZmPSY2(LOC109940986)
81%
83%
82%
82%
79%
79%
85%
84%
0.050
ZmBCH(LOC103631225)
zmBCH1(LOC732775)
SiBCH1(Si9g54300)
OsBCH2(LOC4349224)
OsBCH2'(LOC4331460)
OsBCH2''(LOC4336753)
SiBCH2(Si7g21990)
ZmBCH'(LOC100281693)
ZmBCH3(LOC732825)
AtBCH1(AT4G25700)
AtBCH2(AT5G52570)
80%
83%
80%
78%
87%
85%
85%
80%
82%
0.050
SiZEP1(Si7g13140)
ZmZEP(LOC100285076)
SiZEP2(Si7g13100)
OsZEP(LOC4335984)
AtZEP(AT5G67030)
83%
87%
85%
0.050

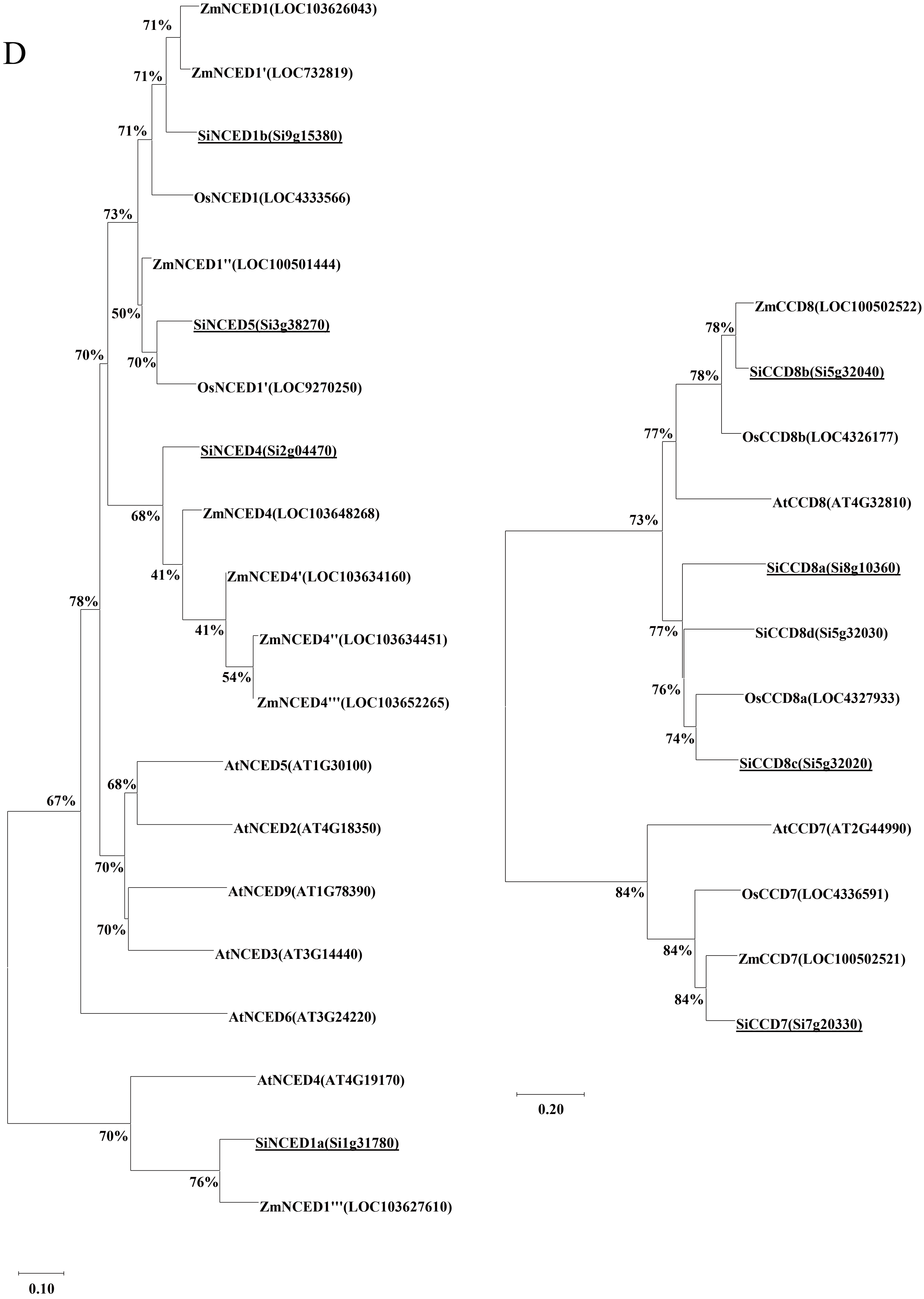
D
ZmNCED1(LOC103626043)
ZmNCED1'(LOC732819)
SiNCED1b(Si9g15380)
OsNCED1(LOC4333566)
ZmNCED1''(LOC100501444)
SiNCED5(Si3g38270)
OsNCED1'(LOC9270250)
SiNCED4(Si2g04470)
ZmNCED4(LOC103648268)
ZmNCED4'(LOC103634160)
ZmNCED4''(LOC103634451)
ZmNCED4'''(LOC103652265)
AtNCED5(AT1G30100)
AtNCED2(AT4G18350)
AtNCED9(AT1G78390)
AtNCED3(AT3G14440)
AtNCED6(AT3G24220)
AtNCED4(AT4G19170)
SiNCED1a(Si1g31780)
ZmNCED1'''(LOC103627610)
71%
71%
71%
73%
50%
70%
70%
68%
41%
78%
41%
54%
68%
67%
70%
70%
70%
76%
0.10
ZmCCD8(LOC100502522)
SiCCD8b(Si5g32040)
OsCCD8b(LOC4326177)
AtCCD8(AT4G32810)
SiCCD8a(Si8g10360)
SiCCD8d(Si5g32030)
OsCCD8a(LOC4327933)
SiCCD8c(Si5g32020)
AtCCD7(AT2G44990)
OsCCD7(LOC4336591)
ZmCCD7(LOC100502521)
SiCCD7(Si7g20330)
78%
78%
77%
73%
77%
76%
74%
84%
84%
84%
0.20

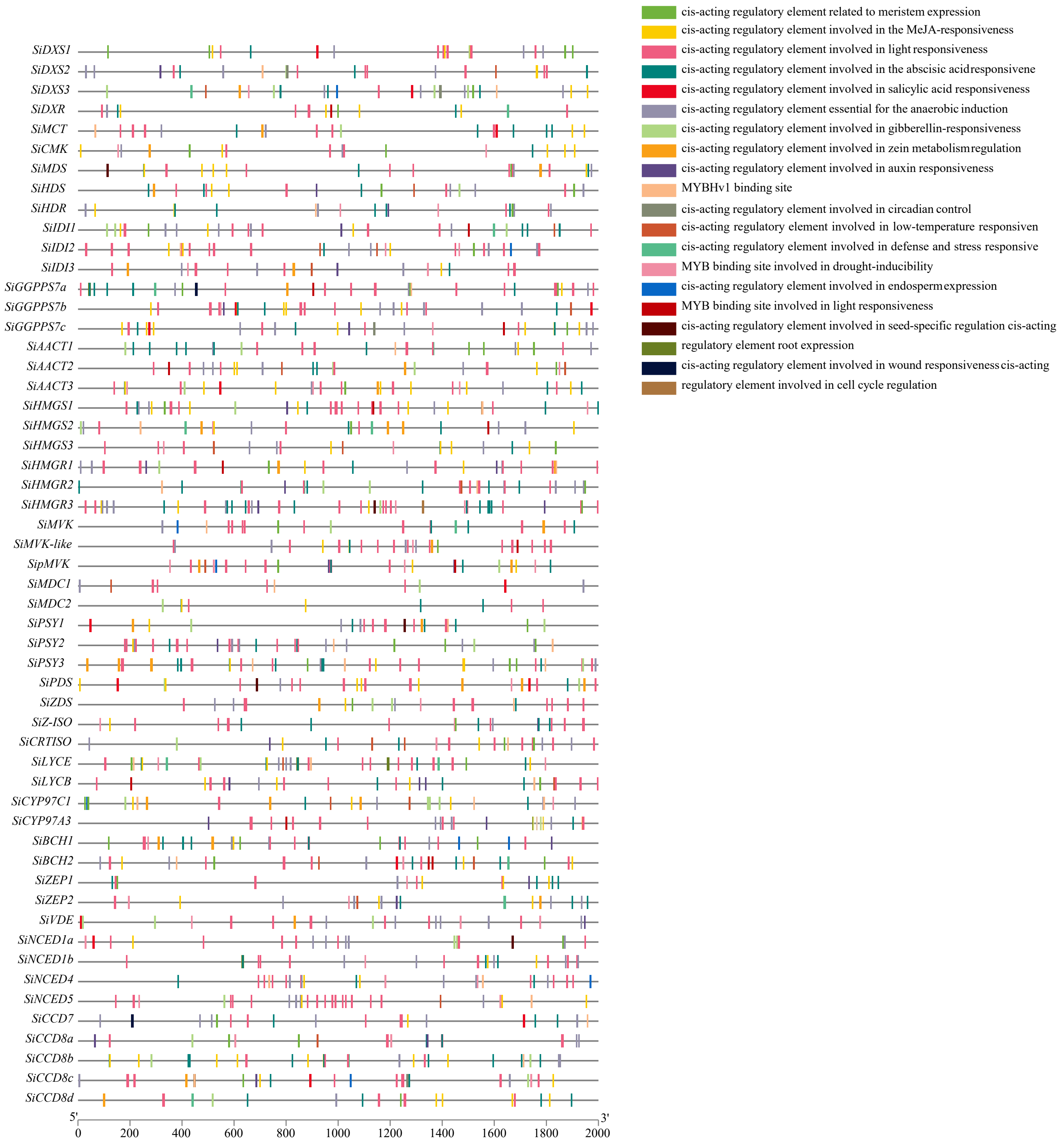

Figure S5. Analysis of cis-acting elements upstream of the promoter of carotenoid metabolism genes.

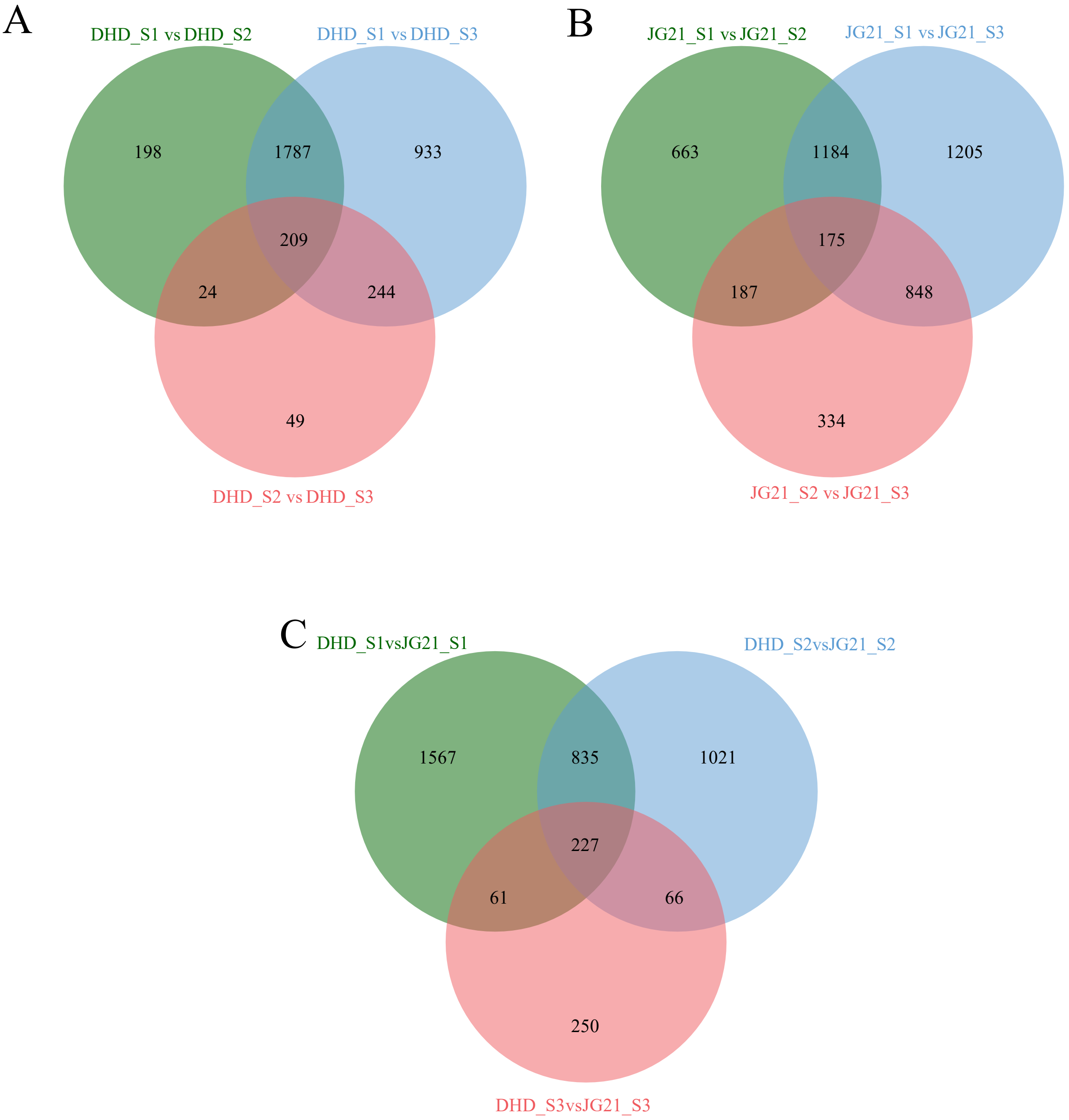

Figure S6. Analysis of differentially expressed genes. (A) Analysis of differentially expressed genes at the three developmental stages of DHD. (B) Analysis of differentially expressed genes at the three developmental stages of JG21. (C) Analysis of differentially expressed genes at DHD and JG21 in different developmental stages.

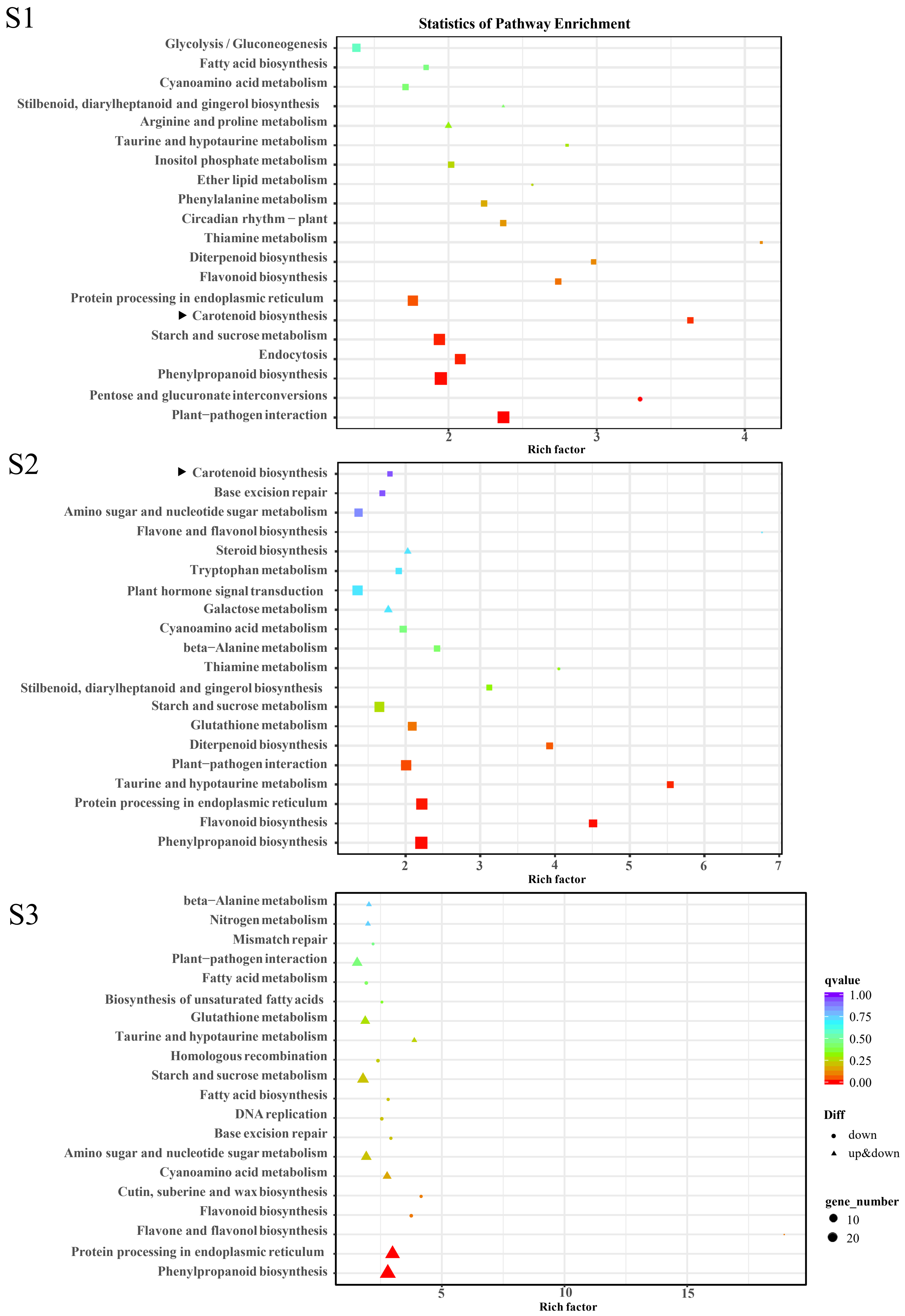

Figure S7. KEGG enrichment analysis of differentially expressed genes in of DHD and JG21 at different grain development stages.

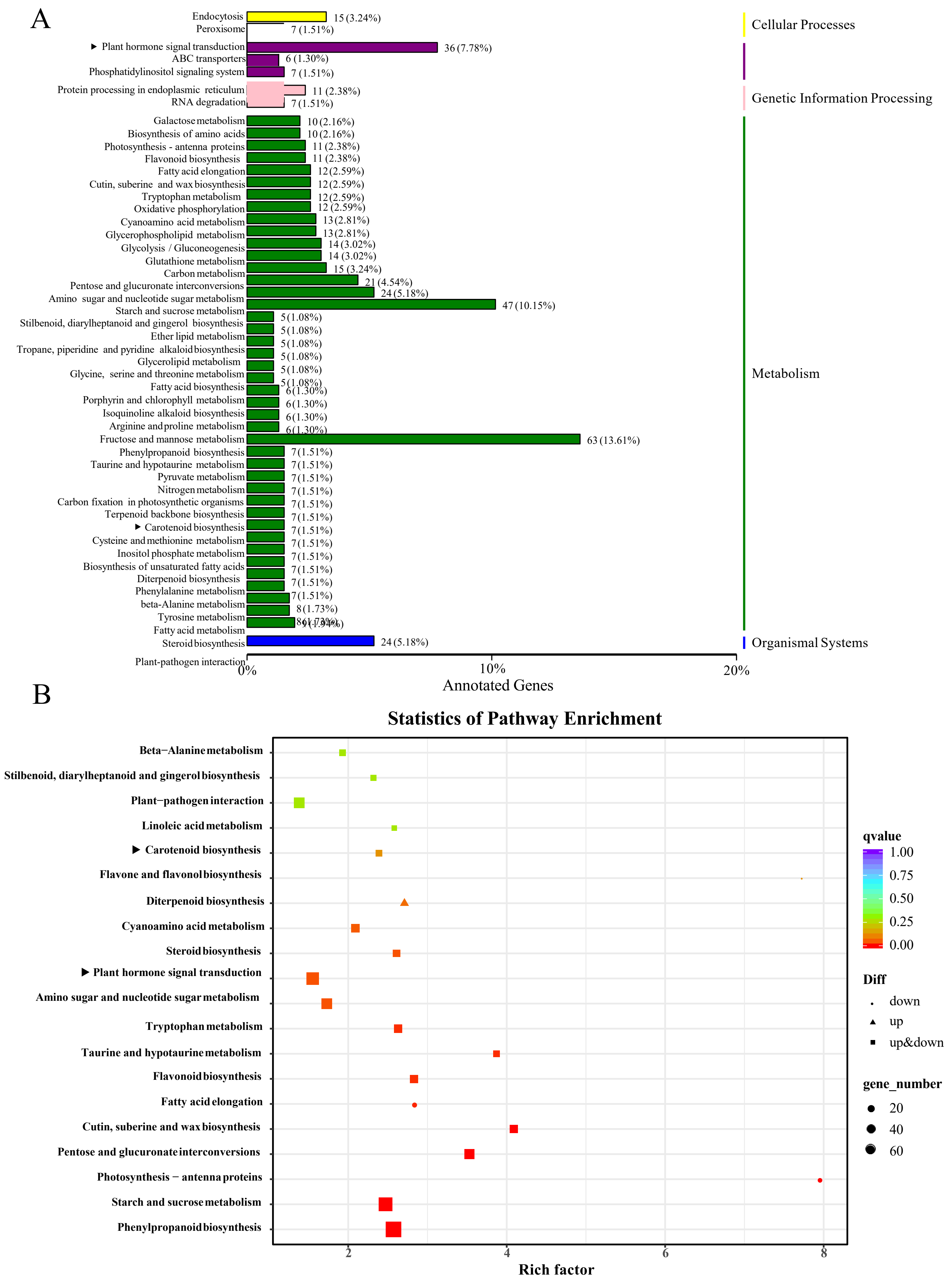

Figure S8. GO and KEGG enrichment analyses of differentially expressed genes at the early and late stages of DHD grain development. (A) GO enrichment analysis. (B) KEGG enrichment analysis.

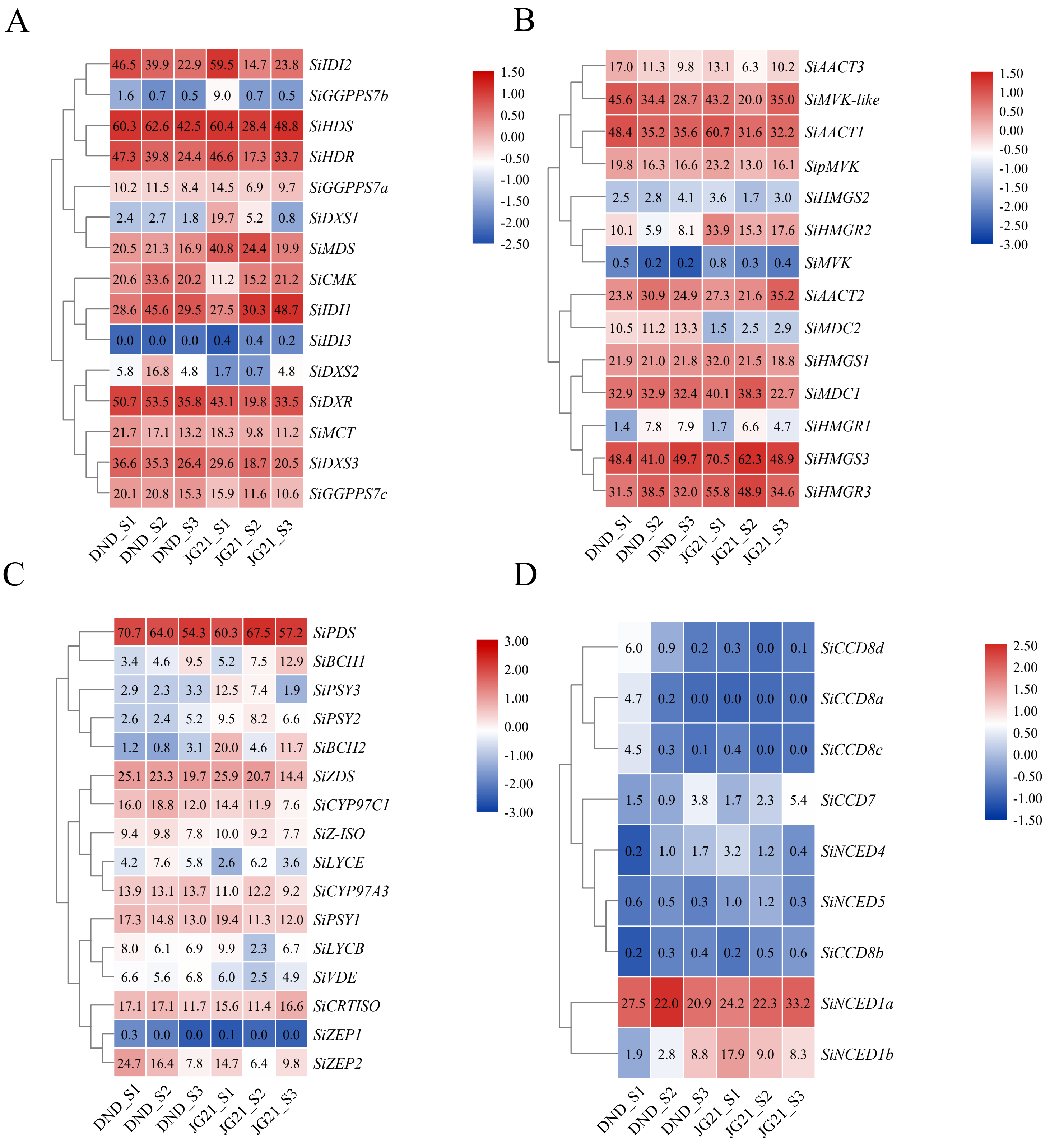

Figure S9. Expression patterns of genes related to carotenoid metabolism in DHD and JG21 at different grain developmental stages. (A) MEP pathway. (B) MVA pathway. (C) Carotenoid biosynthetic pathway. (D) Carotenoid degradation pathway.

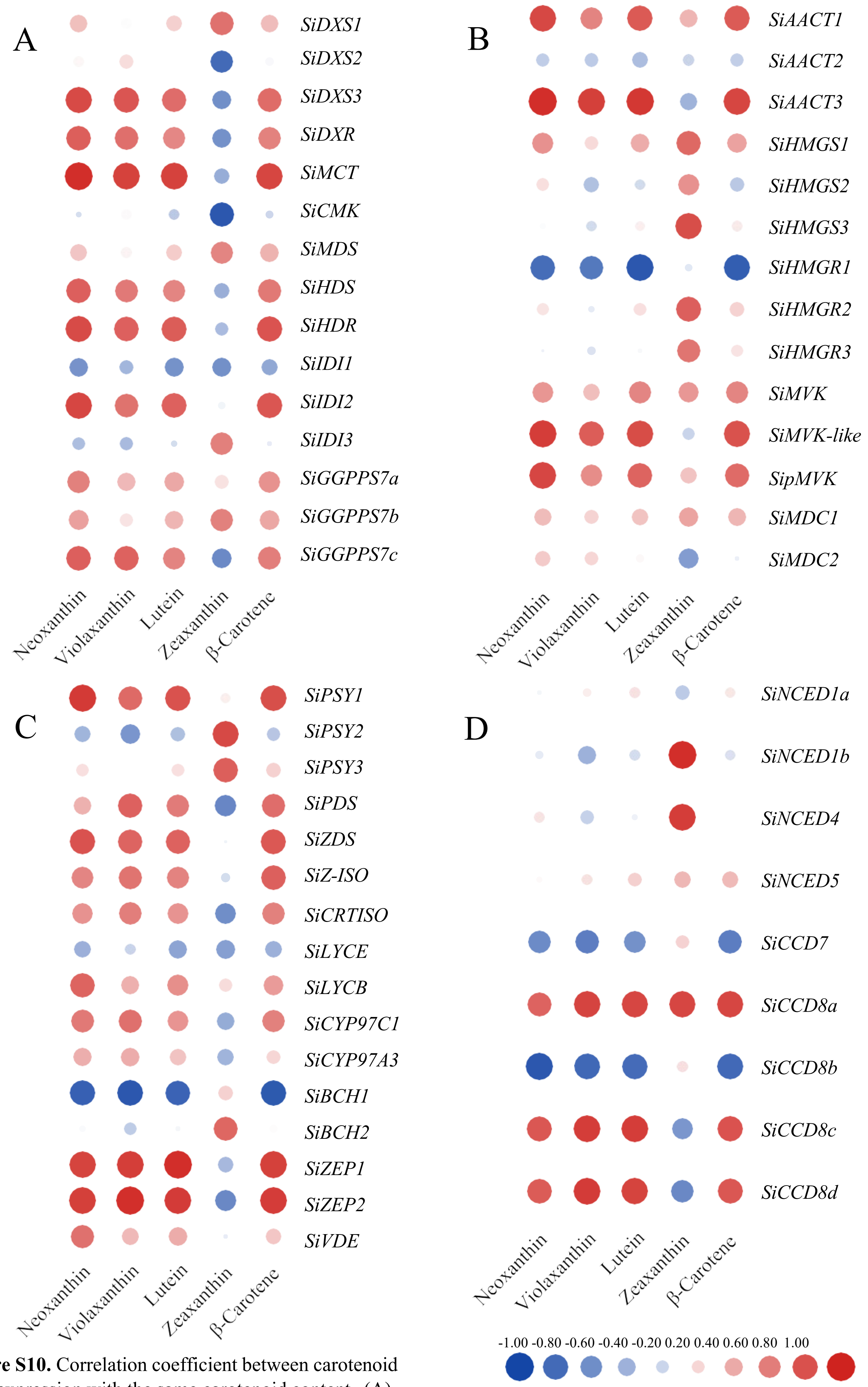

Figure S10. Correlation coefficient between carotenoid gene expression with the same carotenoid content. (A) MEP pathway.
(B) MVA pathway. (C) Carotenoid biosynthetic pathway.
(D) Carotenoid degradation pathway.

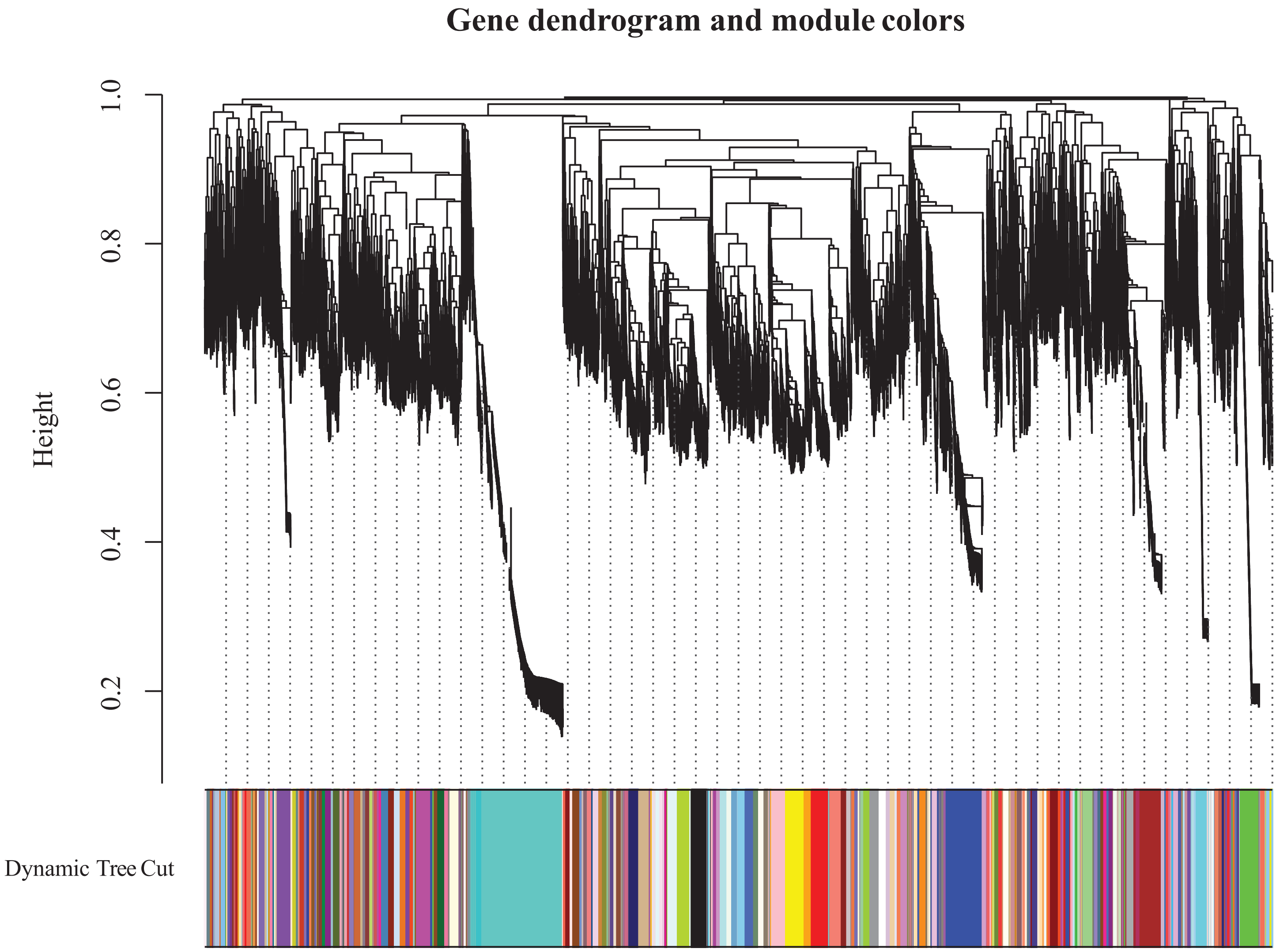

Figure S11. Gene dendrogram and coexpression network module colors.

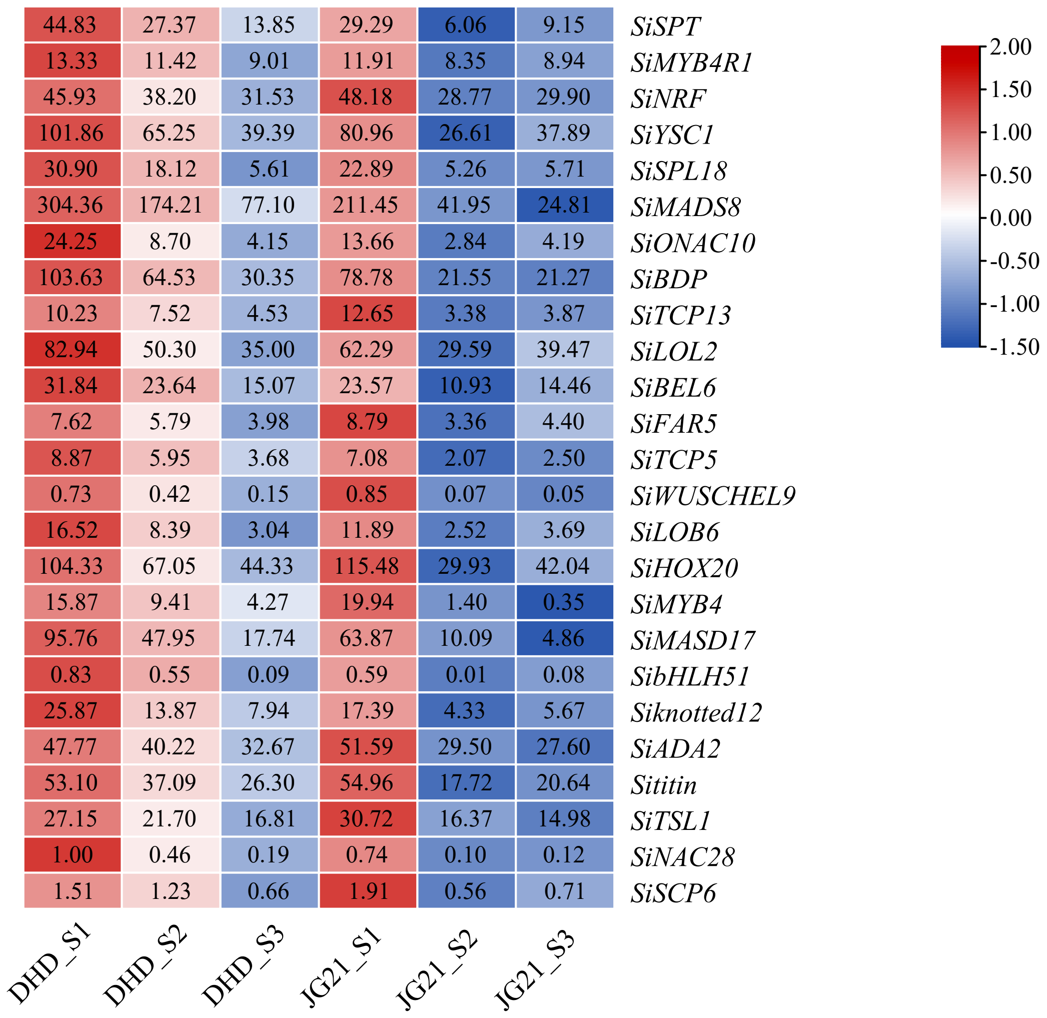

Figure S12. Expression patterns of transcription factor related to carotenoid metabolism in DHD and JG21 at different grain developmental stages.

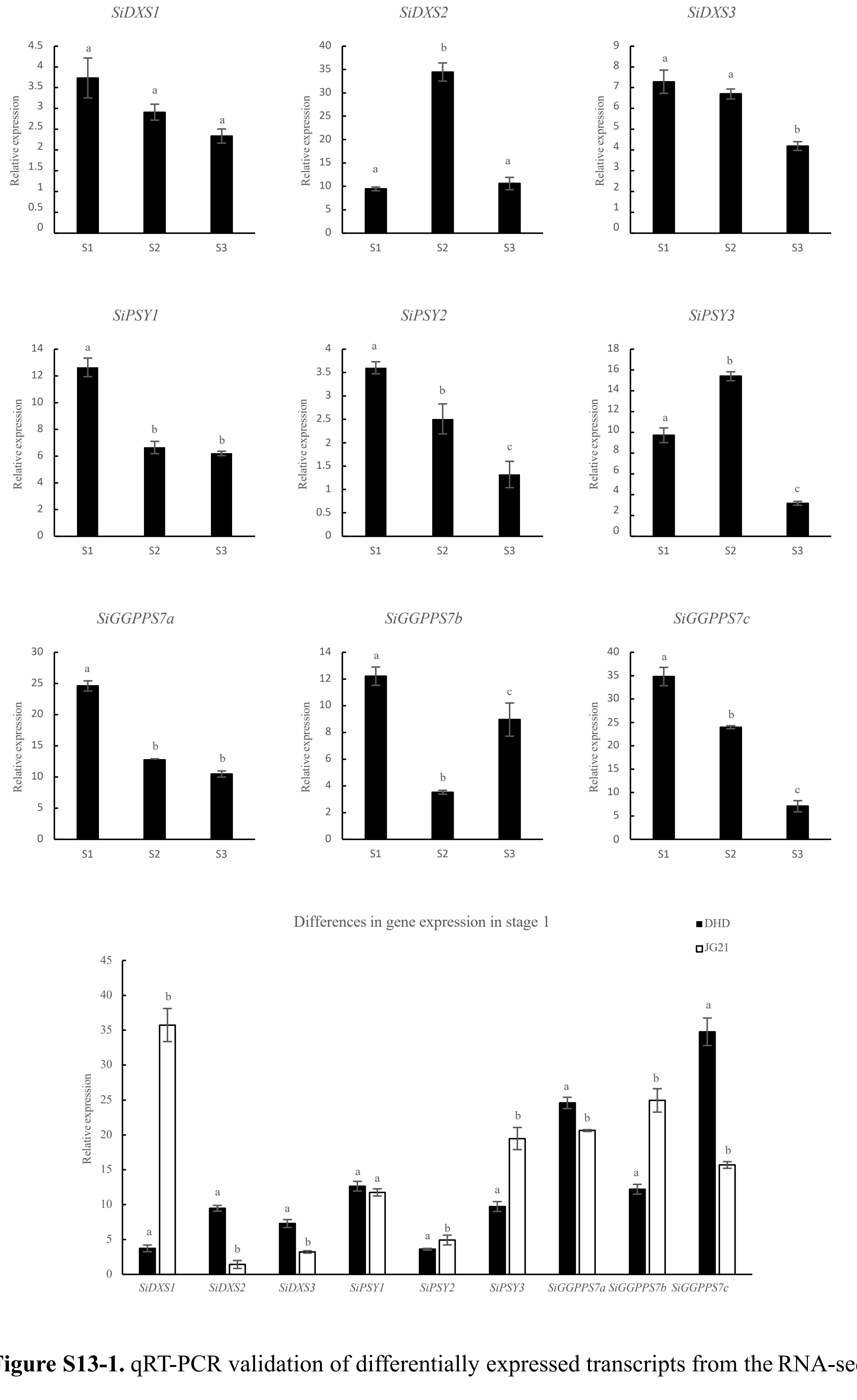

Figure S13-1. qRT-PCR validation of differentially expressed transcripts from the RNA-seq analysis.

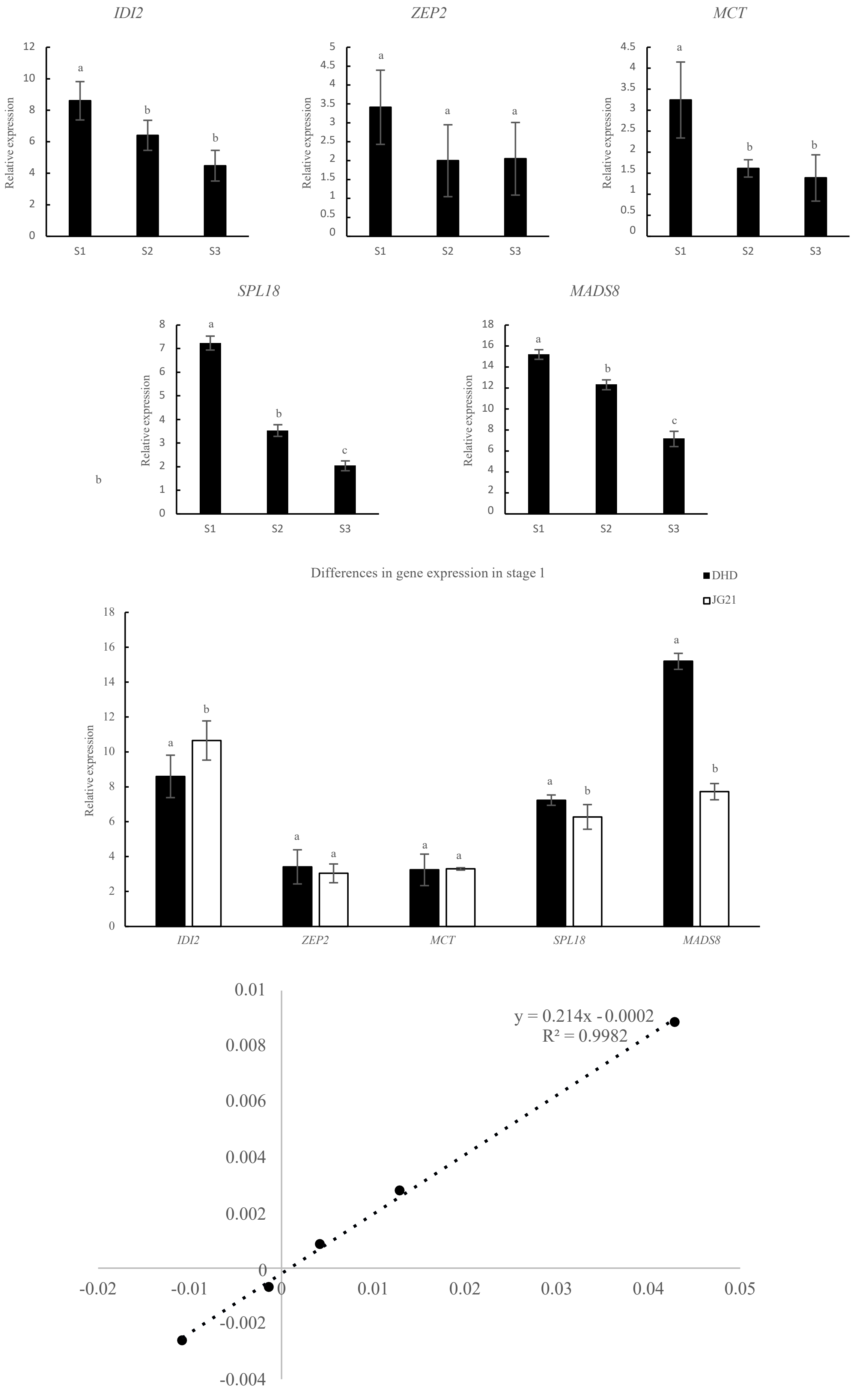

Figure S13-2. qRT-PCR validation of differentially expressed transcripts from the RNA-seq analysis.
